# RNF111-facilitated neddylation potentiates cGAS-mediated antiviral innate immune response

**DOI:** 10.1371/journal.ppat.1009401

**Published:** 2021-03-15

**Authors:** Chenhui Li, Lele Zhang, Dong Qian, Mingxing Cheng, Haiyang Hu, Ze Hong, Ye Cui, Huansha Yu, Quanyi Wang, Juanjuan Zhu, Wei Meng, Jin-fu Xu, Yi Sun, Peng Zhang, Chen Wang

**Affiliations:** 1 State Key Laboratory of Natural Medicines, School of Life Science and Technology, China Pharmaceutical University, Nanjing, Jiangsu, China; 2 Shanghai Pulmonary Hospital, Tongji University School of Medicine, Shanghai, China; 3 Division of Immunology, The Boston Children’s Hospital, Department of Pediatrics, Harvard Medical School, Boston, Massachusetts, United States of America; 4 Cancer Institute of the 2^nd^ affiliated hospital and Institute of Translational Medicine, Zhejiang University School of Medicine, Hangzhou, Zhejiang, China; The Ohio State University, UNITED STATES

## Abstract

The cytosolic DNA sensor cyclic GMP-AMP (cGAMP) synthetase (cGAS) has emerged as a fundamental component fueling the anti-pathogen immunity. Because of its pivotal role in initiating innate immune response, the activity of cGAS must be tightly fine-tuned to maintain immune homeostasis in antiviral response. Here, we reported that neddylation modification was indispensable for appropriate cGAS-STING signaling activation. Blocking neddylation pathway using neddylation inhibitor MLN4924 substantially impaired the induction of type I interferon and proinflammatory cytokines, which was selectively dependent on Nedd8 E2 enzyme Ube2m. We further found that deficiency of the Nedd8 E3 ligase *Rnf111* greatly attenuated DNA-triggered cGAS activation while not affecting cGAMP induced activation of STING, demonstrating that Rnf111 was the Nedd8 E3 ligase of cGAS. By performing mass spectrometry, we identified Lys231 and Lys421 as essential neddylation sites in human cGAS. Mechanistically, Rnf111 interacted with and polyneddylated cGAS, which in turn promoted its dimerization and enhanced the DNA-binding ability, leading to proper cGAS-STING pathway activation. In the same line, the *Ube2m* or *Rnf111* deficiency mice exhibited severe defects in innate immune response and were susceptible to HSV-1 infection. Collectively, our study uncovered a vital role of the Ube2m-Rnf111 neddylation axis in promoting the activity of the cGAS-STING pathway and highlighted the importance of neddylation modification in antiviral defense.

## Author summary

The Cyclic GMP-AMP (cGAMP) synthase (cGAS) is a critical cytosolic DNA sensor by monitoring pathogens-derived DNA, while its aberrant activation leads to tissue damage. Thus, the activity of cGAS must be tightly regulated to keep immune homeostasis. Several post-translational modifications have been demonstrated to be important in fine-tuning activities of cGAS. However, the mechanism underlying how cGAS is precisely regulated remains not fully understood. In this study, we uncovered that cGAS was poly-neddylated by the Ube2m-Rnf111 axis, and the poly-NEDD8 chains played a role as nexus in linking the second cGAS, leading to the appropriate dimerization and the subsequent activation of cGAS. Our study revealed a novel and critical role of neddylation in the regulation of the cGAS-STING signaling pathway and provided a new perspective for restricting the infection of DNA pathogens.

## Introduction

Innate immunity plays pivotal roles in pathogen defense and tissue repair. It is mainly achieved by pattern recognition receptors (PRRs), which detect pathogen associated molecular patterns (PAMPs, from pathogens) and danger-associated molecular patterns (DAMPs, from host) to evoke instant activation of innate immunity and the subsequent adaptive immunity [1–3]. Nucleic acids have been defined as a fundamental mechanism to elicit innate immunity by a heterogeneous group of PRRs. The cyclic GMP-AMP (cGAMP) synthase cGAS is a key cytosolic DNA sensor [4,5]. After binding with cytosolic DNA, cGAS is activated and converts ATP and GTP into cGAMP, which then activates stimulator of interferon genes (STING) to trigger immune responses. The cGAS-STING pathway is an important component of the innate immunity against pathogen infection, with the fact that cGAS or STING deficiency mice are unable to induce type I interferon and susceptible to DNA viruses or *Listeria monocytogenes* [6,7]. However, the inappropriate activation of the cGAS–STING pathway may initiate autoimmune diseases or exacerbate non-infectious inflammation, so its activation should be tightly regulated within a proper scope [8–10].

Post-translational modifications such as ubiquitylation, and ubiquitylation-like sumoylation have been reported to be critical for the regulation of the cGAS-STING pathway [5,11,12]. Nevertheless, the roles of other ubiquitin-like proteins in regulating the cGAS-STING pathway remain elusive. NEDD8 (neural precursor cell expressed, developmentally downregulated 8) is a ubiquitin-like protein, which is covalently conjugated to the substrates in a process termed as neddylation. The typical neddylation transfer process relies on the enzymatic cascade of NEDD8-activating enzyme (E1, a heterodimer of APPBP1 and UBA3), NEDD8-conjugating enzyme (E2, UBE2M or UBE2F) and NEDD8-ligase (E3) [13]. Neddylation is involved in various physiological and pathological processes, including the development of inflammatory and autoimmune diseases [14,15]. It has long been known neddylation can be inhibited during pathogenic infection. The deaminase enzymes of several bacteria can deamidate Gln40 in NEDD8, which abolishes the activity of neddylated substrate [16,17]. However, how NEDD8 modulates the activity of antipathogenic immune signaling is still largely unknown.

In this report, we investigated the role of neddylation pathway in modulating the cGAS-STING signaling pathway. We found cGAS is subjected to neddylation modification, and the neddylation of cGAS enhanced its ability to dimerization and strengthened its DNA-binding ability. Importantly, blockage of neddylation by using either neddylation inhibitor MLN4924 or deficiency of Nedd8 E2 enzyme *Ube2m* or E3 *Rnf111* substantially attenuated activation of the cGAS-STING pathway induced by double-stranded DNA (dsDNA). Consequently, conditional-knockout (cKO) mice with deletion of *Ube2m* or *Rnf111* in myeloid lineage (by LyzM-Cre) were more susceptible to DNA viruses, as compared to the control mice, largely due to severely disrupted innate immune response. Collectively, our results demonstrated that neddylation modification by the Ube2m-Rnf111 axis is indispensable for cGAS activation and immunity against pathogens.

## Results

### Inhibition of neddylation impairs dsDNA-induced activation of the cGAS-STING pathway

**Fig 1:**
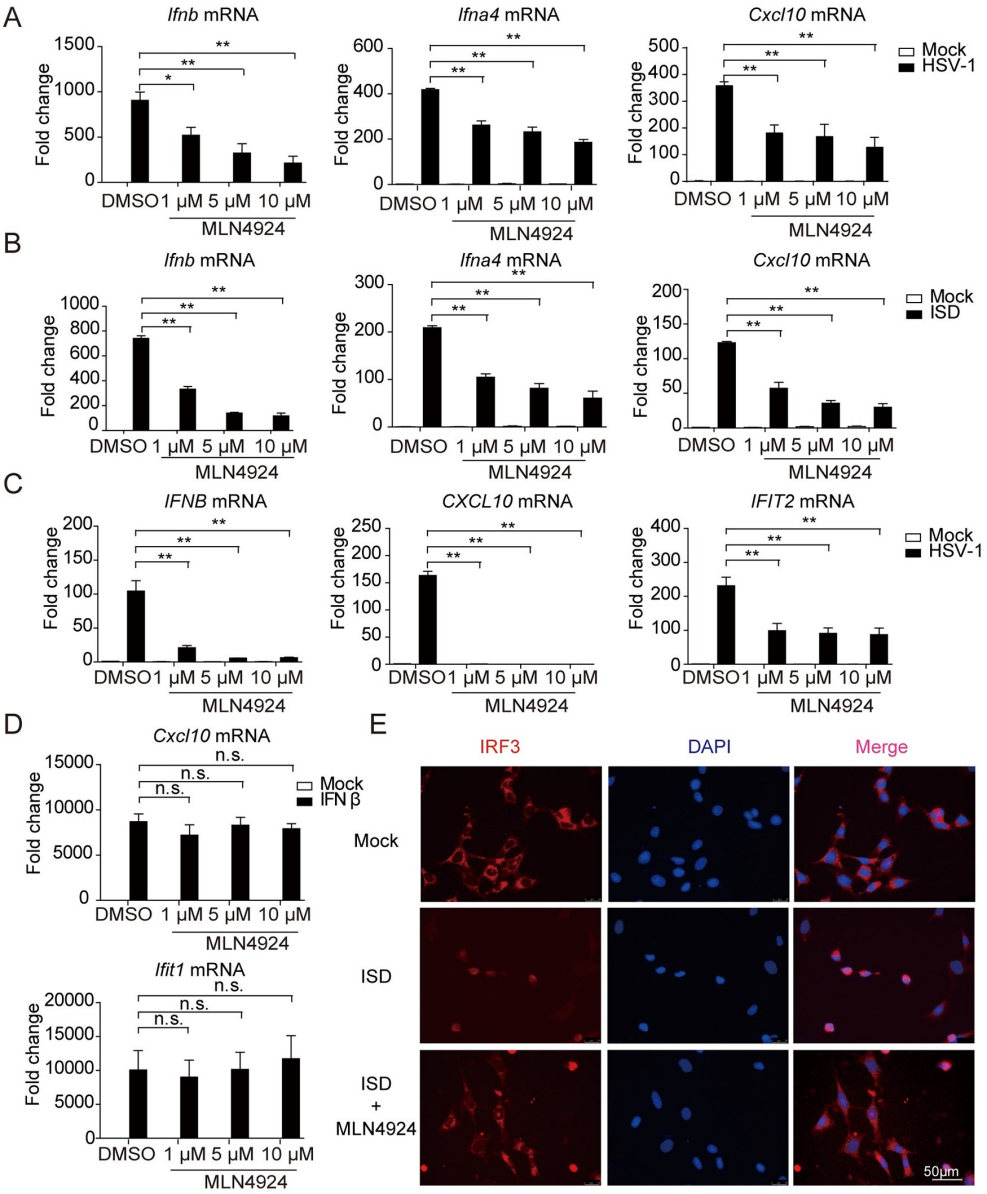
Neddylation facilitates the DNA-triggered signaling pathway. (A and B) MEFs were pretreated with MLN4924 for 2h, followed by stimulation with HSV-1 (MOI = 1) or ISD for 6h, the transcription of *Ifnb*, *Ifna4* and *Cxcl10* were measured by qRT-PCR. (C) THP-1 cells were pretreated with MLN4924 for 2h and then treated with HSV-1 (MOI = 1) for 6h, the transcription of *IFNB*, *CXCL10* and *IFIT2* were measured by qRT-PCR. (D) MEFs were pretreated with MLN4924 for 2h and then treated with IFNβ, the induction of *Cxcl10* and *Ifit1* were measured by qRT-PCR. (E) MEFs were pretreated with MLN4924 for 2h, followed by stimulation with ISD for 6h, then fixed and stained with an antibody specific for IRF3 and imaged by confocal microscopy. Scale bars represent 50 μm. Graphs are presented as means ± SEM, data are representative of three independent experiments, *P <0.05; **P <0.01 (One-way ANOVAs followed by Tukey’s post hoc test).

To determine whether neddylation modulates the cGAS-STING pathway, we first examined potential changes in various immune responses triggered by cGAS-STING activation upon blockage of neddylation modification in human and mouse cells. MLN4924, a small molecule inhibitor of NEDD8 activating enzyme, known to block the entire process of neddylation *in vivo* was used [14,18]. MLN4924 pretreatment severely decreased the levels of *Ifnb*, *Ifna4* and *Cxcl10* mRNAs induced by HSV-1 or ISD in a dose-dependent manner in mouse embryonic fibroblasts (MEFs) (Fig 1A and 1B). Similarly, MLN4924 pretreatment also significantly attenuated *IFNB*, *CXCL10* and *IFIT2* production, triggered by HSV-1 in human THP-1 cells, while not affecting *Cxcl10* and *Ifit1* expression induced by IFNβ in MEFs (Fig 1C and 1D). Consistently, confocal microscopy also showed that the nuclear translocation of IRF3 was inhibited by MLN4924 (Fig 1E). To further confirm these results, we measured the potential changes of innate immune response after knockdown of *Uba3*, the catalytic subunit of NEDD8 activating enzyme to which MLN4924 inhibits [13,19]. Similar to the results obtained from pharmacological approach using MLN4924, this genetic approach remarkably decreased the transcription of *Ifnb*, *Ifna4* and *Cxcl10* genes induced by HSV-1 or ISD in MEFs (S1A Fig), so did the transcription of *Ifnb*, *Ifna4* and *Cxcl10* genes induced by HT-DNA in L929 cells (S1B Fig). The efficiency of siRNA-mediated knockdown of *Uba3* mRNA is shown in S1C Fig. Given neddylation is a reversible post-translational modification [13], and Senp8 was a newly identified deneddylase whose inhibition enhanced neddylation modification on substrates [20]. Indeed, knockdown of *Senp8* remarkably enhanced the transcription of *Ifnb*, *Ifna4* and *Cxcl10* genes after ISD and HT-DNA stimulations (S1D and S1E Fig). Taken together, these observations demonstrated that neddylation is required for dsDNA-induced activation of the cGAS-STING pathway.

### Depletion of *Ube2m* but not *Ube2f* attenuates dsDNA-induced innate immune response

**Fig 2:**
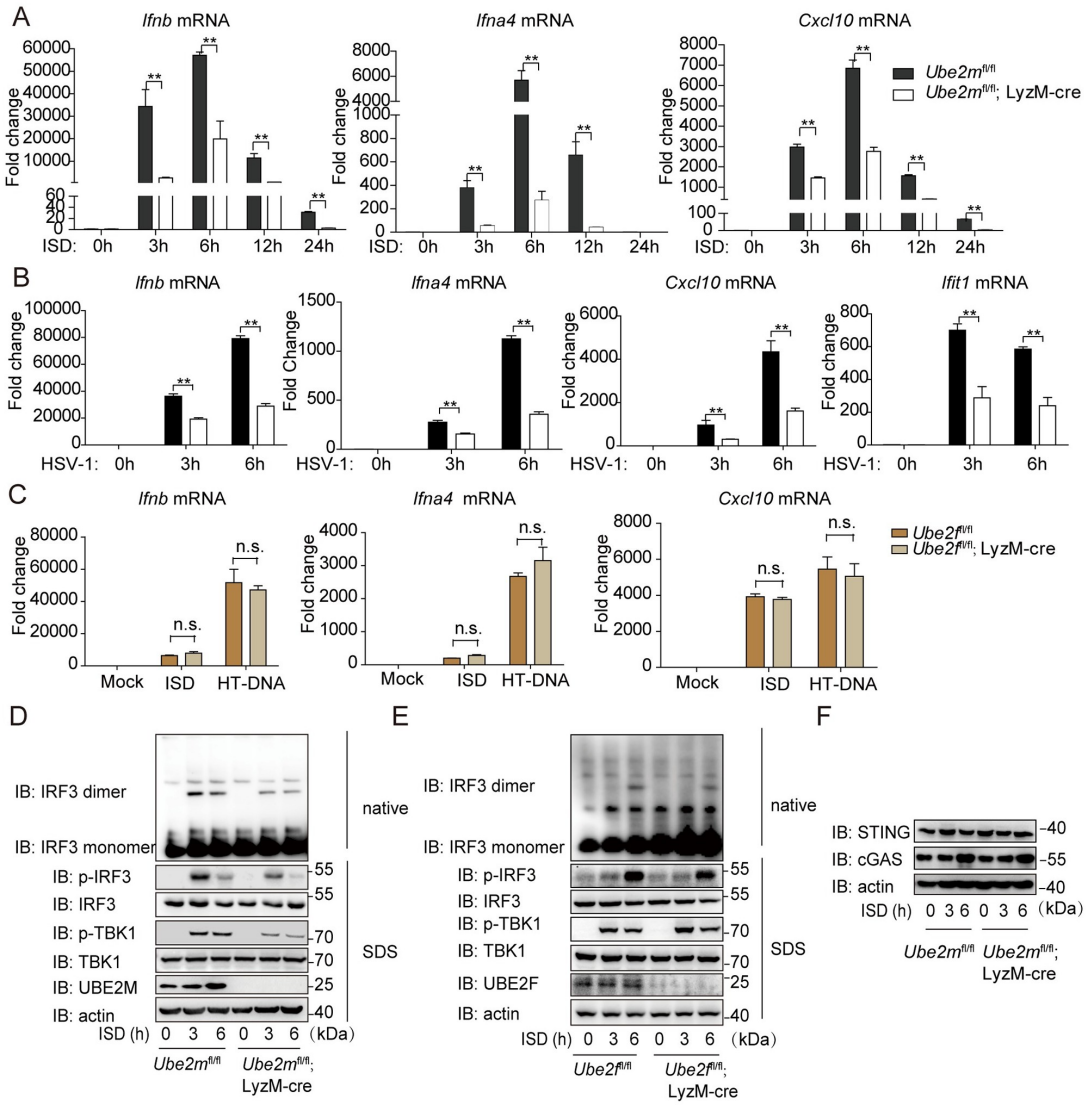
Depletion of Ube2m but not Ube2f attenuates dsDNA-induced innate immune response. (A) Effects of *Ube2m* deficiency on the expression of *Ifnb*, *Ifna4* and *Cxcl10* after ISD stimulation for indicated hours in BMDM. (B) Effects of *Ube2m* deficiency on the expression of *Ifnb*, *Ifna4*, *Cxcl10* and *Ifit1* after HSV-1 (MOI = 1) stimulation in BMDM. (C) Effect of *Ube2f* deficiency on the expression of *Ifnb*, *Ifna4* and *Cxcl10* after ISD and HT-DNA stimulation in BMDM. (D) Effects of *Ube2m* deficiency on the phosphorylation of TBK1/IRF3 and the dimerization of IRF3 after ISD stimulation for the indicated time. (E) Effects of *Ube2f* deficiency on phosphorylation of TBK1/IRF3 and the dimerization of IRF3 after ISD stimulation for the indicated time. (F) Effects of *Ube2m* deficiency on the protein level of cGAS and STING. Graphs are presented as means ± SEM, data are representative of three independent experiments, *P <0.05; **P <0.01 (One-way ANOVAs followed by Tukey’s post hoc test).

In mammalian cells, there are only two family members of neddylation E2 enzymes, UBE2M and UBE2F [21]. We next investigated which E2 mediated the cGAS-STING activation. To this end, we generated *Ube2m*^fl/fl^ and *Ube2f*^fl/fl^ mice [22] and then crossed with the LyzM-Cre mice, leading to tissue specific deletion of either of E2 in the myeloid lineage, respectively. The quantitative real-time PCR (qRT-PCR) analysis showed the transcription of *Ifnb*, *Ifna4* and *Cxcl10* genes were significantly down-regulated in BMDM from *Ube2m*^fl/fl^; LyzM-cre mice from 3 to 24 hours after ISD stimulation (Fig 2A). Moreover, *Ube2m* deficiency decreased the transcription of *Ifnb*, *Ifna4*, *Cxcl10* and *Ifit1* induced by HSV-1 infection (Fig 2B). In the same vein, the ISD-induced phosphorylation of TBK1, IRF3, p65 and IκBα, as well as dimerization of IRF3 were strongly inhibited in *Ube2m* deficient BMDM, as compared to their counterparts (Fig 2D and S2C Fig). In contrast, *Ube2m* deficiency increased Poly(I:C) or Sendai virus (SeV) induced transcription of *Ifnb* and *Ifna4* genes (S2A and S2B Fig). These results indicated that Ube2m specifically facilitated cytosolic DNA and DNA virus triggered antiviral pathway.

By contrast, *Ube2f* depletion in myeloid lineage influenced neither the mRNA levels of *Ifnb*, *Ifna4* and *Cxcl10* induced by ISD and HT-DNA (Fig 2C), nor the phosphorylation of TBK1 and phosphorylation/ dimerization of IRF3 induced by ISD (Fig 2E). To be mentioned, *Ube2m* deficient did not affect the protein level of cGAS and STING compared to their counterparts (Fig 2F). Collectively, these data demonstrated that Ube2m, but not Ube2f, specifically facilitated the activity of the cGAS-STING pathway.

### Rnf111 is essential for the innate immune response to DNA

**Fig 3:**
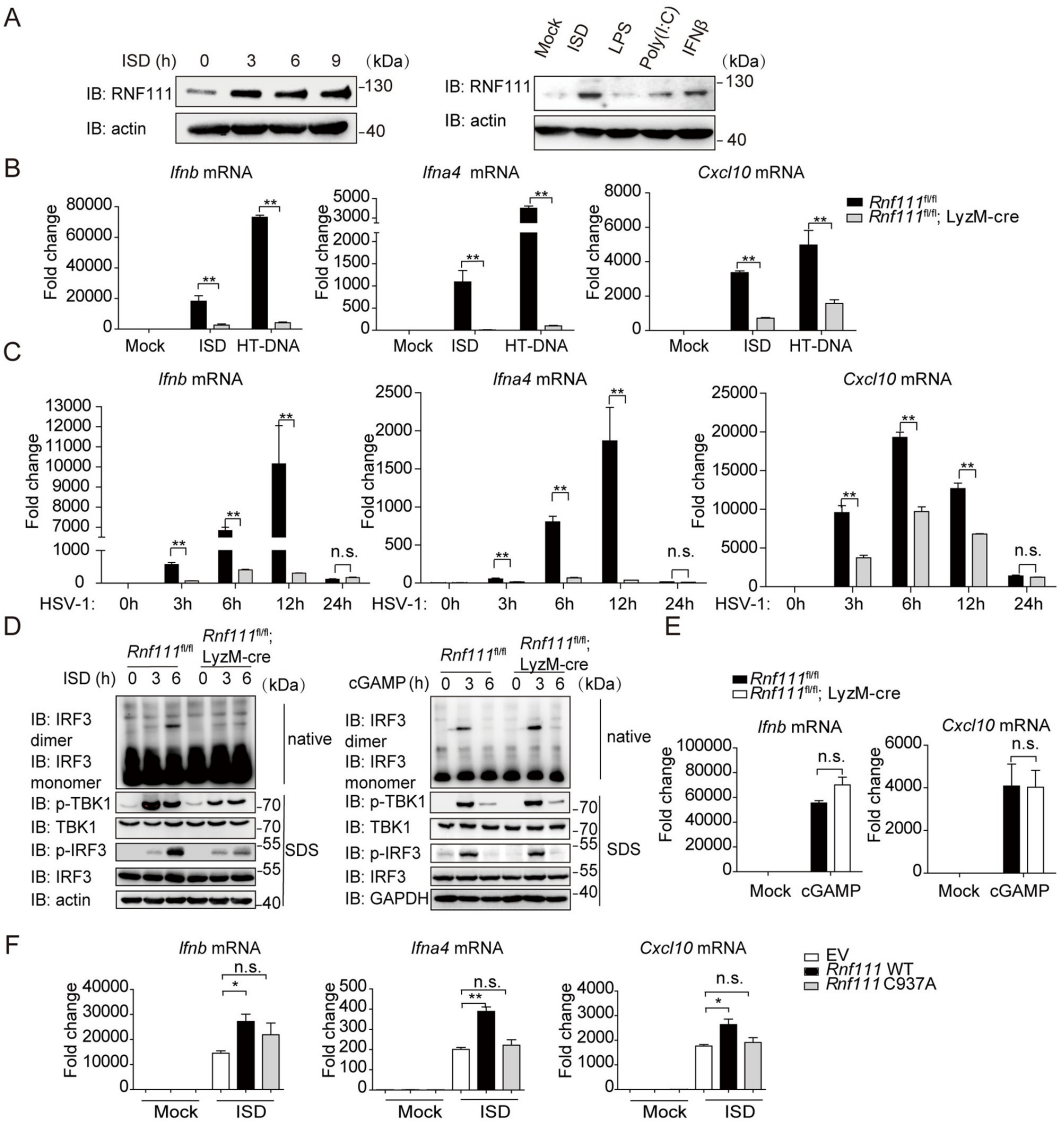
Rnf111 facilitates dsDNA-triggered signaling. (A) dsDNA stimulation promoted the expression of Rnf111 in MEFs (left) and BMDM (right). (B) Effect of *Rnf111* deficiency on the expression of *Ifnb*, *Ifna4* and *Cxcl10* after ISD and HT-DNA stimulation for indicated hours in BMDM. (C) Effect of *Rnf111* deficiency on the expression of *Ifnb*, *Ifna4* and *Cxcl10* after HSV-1 (MOI = 0.5) infection in BMDM. (D) Effects of *Rnf111* deficiency on IRF3 dimerization and phosphorylation of TBK1/IRF3 after ISD (left) or cGAMP (right) stimulation for the indicated time. (E) Effect of *Rnf111* deficiency on the expression of *Ifnb* and *Cxcl10* after cGAMP stimulation for 3h in BMDM. (F) *Rnf111* deficiency BMDM were transfected with 100 ng of empty vector (EV, pcDNA4.0) or plasmids for the expression of wild-type His-Rnf111 or His-Rnf111 C937A. 48h after transfection, BMDM were stimulated with ISD for 6h, the transcription of *Ifnb*, *Ifna4* and *Cxcl10* were measured by qRT-PCR. Graphs are presented as means ± SEM, data are representative of three independent experiments, *P <0.05; **P <0.01 (One-way ANOVAs followed by Tukey’s post hoc test).

Having established the critical role of Ube2m in DNA elicited innate immune response, we then investigated which NEDD8 E3 ligase was responsible for this process. To date, several NEDD8 E3 ligases have been identified, including RBX1, RBX2, c-CBL, DCN1 and RNF111 [21,23–25]. We screened a library of small interfering RNAs (siRNAs) targeting each of these NEDD8 E3 ligases, and measured their effects on innate immune response after HT-DNA stimulation. The result showed that siRNA targeting *Rnf111* impaired the transcription of *Ifnb* specifically (S3A Fig). Interestingly, we noticed that DNA stimulation increased the protein levels of Rnf111 in MEFs and BMDM (Fig 3A) while not affecting the transcription of *Rnf111* (S3B Fig). To confirm the potential function of Rnf111 on the cGAS-STING signaling, we knocked down *Rnf111* with siRNAs in different cells. The results showed that knockdown of *Rnf111* strongly decreased the transcription of *Ifnb*, *Ifna4* and *Cxcl10* induced by ISD in MEFs (S3C Fig). Similar results were observed when *RNF111* was knocked down in L929 and human foreskin fibroblast (HFF) after HT-DNA stimulation (S3D and S3E Fig). In line with this, knockdown of *Rnf111* remarkable perturbed the phosphorylation of IRF3 and TBK1 in response to ISD stimulation (S3F Fig). The efficiency of siRNA-mediated knockdown of *Rnf111* mRNA was shown in S3G Fig. Notably, knockdown of *Rnf111* affected neither the up-regulation of *Ifnb*, *Ifna4* and *Il6* by CpG-DNA, nor the transcription of *Ifnb*, *Ifna4* and *Cxcl10* induced by cGAMP or c-di-GMP (S3H and S3I Fig), suggesting that Rnf111 might act specifically on the cytosolic DNA sensing pathway by targeting the upstream of STING.

To confirm the above results *in vivo*, we generated *Rnf111*^fl/fl^ mice, and further created myeloid-cell-specific *Rnf111*-deficient mice by crossing with LyzM-cre mice. We confirmed that *Rnf111* was efficiently deleted in BMDM (S4A Fig). *Rnf111* deficiency affected neither the protein level of cGAS and STING, nor the cytoplasmic or nuclear distribution of cGAS (S4B and S4C Fig). Compared with control BMDM, the transcription of *Ifnb*, *Ifna4* and *Cxcl10* induced by ISD, HT-DNA or HSV-1 were significantly impaired in BMDM of *Rnf111*^fl/fl^; LyzM-cre mice (Fig 3B and 3C). By contrast, *Rnf111* deficiency remarkably increased the transcription of *Ifnb*, *Ifna4* and *Cxcl10* induced by Poly (I:C) or SeV (S4D and S4E Fig). These results indicated that Rnf111 specifically facilitated cytosolic DNA and DNA virus triggered antiviral pathway.

The phosphorylation of TBK1, IRF3, p65 and IκBα as well as dimerization of IRF3 were disrupted after ISD stimulation upon *Rnf111* depletion (Fig 3D left and S4F Fig). However, *Rnf111* deficiency did not affect cGAMP induced phosphorylation of TBK1 and phosphorylation/ dimerization of IRF3 and cytokine transcription (Fig 3D right and 3E). Importantly, transfecting wild-type but not the E3-defective mutant *Rnf111* C937A plasmid rescued the defects in DNA-stimulated gene transcription caused by *Rnf111* deficiency (Fig 3F), indicating a causal role of Rnf111 in a manner dependent of its E3 enzymatic activity. These results further demonstrated that Rnf111 was essential to elicit innate immune response to DNA.

RNF111 is also an E3, coupled with UBE2N or UBE2d2α as the E2 for ubiquitination [26,27]. To further exclude the potential influence of ubiquitylation pathway, we repeated the experiments after knocking down *Ube2n* and *Ube2d2a* using siRNAs. The results showed that the deficiency of neither *Ube2n* nor *Ube2d2a* influenced *Ifnb*, *Ifna4* and *Cxcl10* expression induced by ISD (S4G Fig). It has been reported that UBE2M-RNF111 mediated neddylation recruits RNF168 to DNA damage sites [23]. To rule out the possibility of Rnf111 might modulate cGAS through Rnf168, we measured the influence of Rnf168 on the cGAS-STING activation in *Rnf168* deficiency mice [28]. The result showed that knockout of *Rnf168* affected neither the expression of *Ifnb*, *Ifna4* and *Cxcl10*, nor the phosphorylation of TBK1 after induced by HT-DNA or ISD (S5A and S5B Fig). Taken together, these results demonstrated that Rnf111 was essential to elicit innate immune response to DNA in a manner dependent of its E3 ligase activity.

### Rnf111 interacts with cGAS

**Fig 4:**
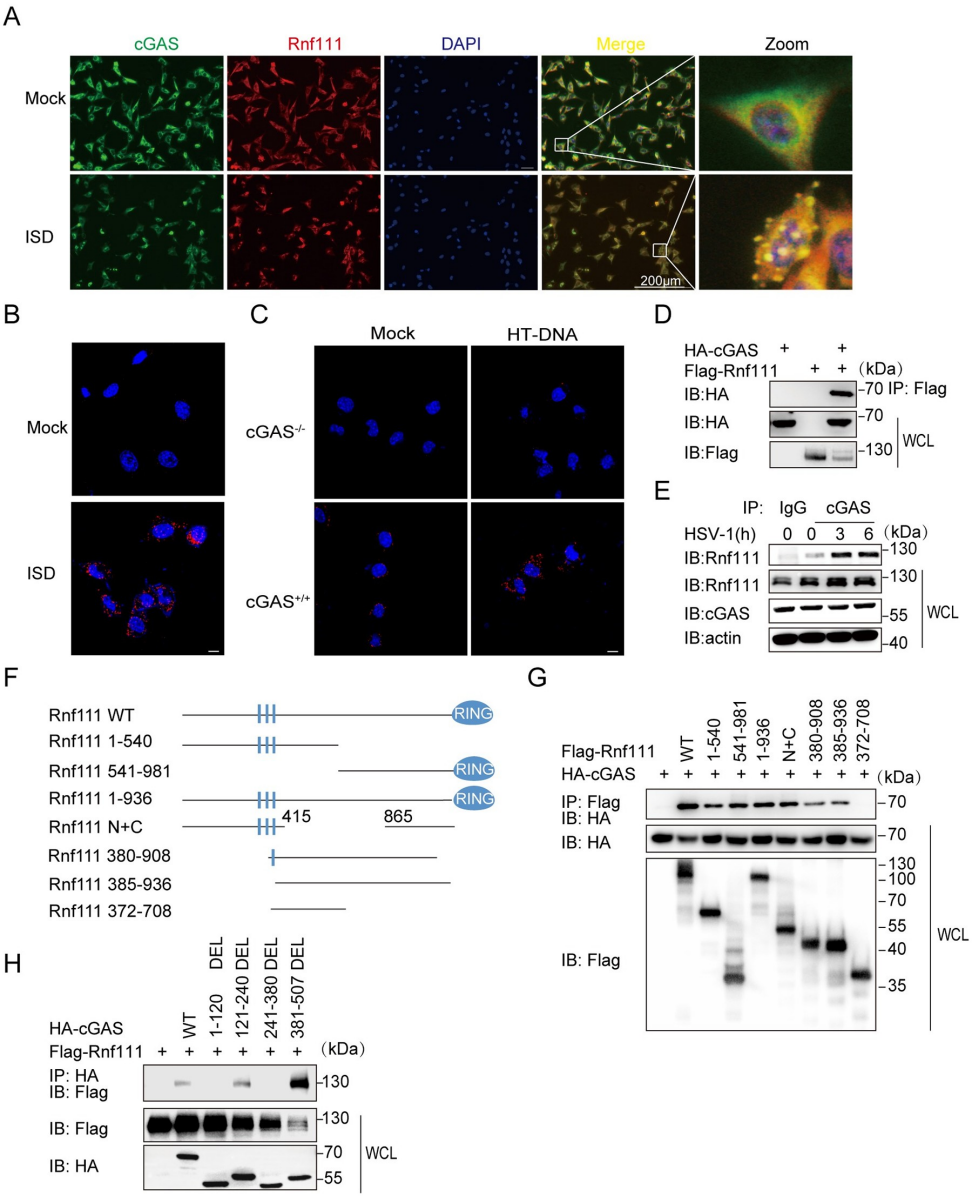
Rnf111 interacts with cGAS. (A) MEFs were stimulated with ISD for 3h, stained with indicated antibodies and imaged by confocal microscopy. Scale bars represent 200 μm. (B) MEFs were stimulated with ISD for 3h, then PLA analysis was applied to detect the interaction between cGAS and Rnf111. Scale bars represent 10 μm. (C) WT or cGAS^-/-^ L929 were stimulated with HT-DNA for 3h, then PLA analysis was applied to detect the interaction between cGAS and Rnf111. Scale bars represent 10 μm. (D) HEK293T cells were transfected with indicated plasmids, 24h after transfection, cell lysis was immunoprecipitated with an anti-Flag antibody and then immunoblotted with indicated antibodies. (E) MEFs were infected with HSV-1 (MOI = 0.5) for the indicated time, cell lysis was immunoprecipitated with an anti-cGAS or IgG antibodies and then immunoblotted with indicated antibodies. (F) Diagrams of Rnf111 truncations used in this paper. (G) HEK293T cells were transfected with indicated plasmids for 24h, then cell lysis was immunoprecipitated with anti-Flag antibody and then immunoblotted with indicated antibodies. (H) HEK293T cells were co-transfected with plasmids of different truncations of cGAS and Rnf111, 24h after transfection, lysates of HEK293T were immunoprecipitated with anti-HA antibody and then immunoblotted with indicated antibodies.

Given that Rnf111 regulated the cGAS-STING pathway and might act in upstream of STING, we then investigated whether Rnf111 could interact with cGAS. Our confocal microscopy results showed that Rnf111 co-localized with cGAS after ISD stimulation (Fig 4A). In addition, proximity ligation assay (PLA) confirmed that Rnf111 interacted with cGAS after ISD stimulation in MEFs (Fig 4B). By contrast, no colocalization was observed in cGAS^-/-^ L929 after HT-DNA stimulation (Fig 4C). To further confirm the colocalization between Rnf111 and cGAS, we cotransfected exogenous cGAS and Rnf111 in HEK293T. The immunoprecipitation (IP) results showed that cGAS interacted with Rnf111 (Fig 4D). Furthermore, we detected endogenous interaction between cGAS and Rnf111 was significantly enhanced upon HSV-1 infection (Fig 4E). We next investigated which domains of each protein mediated their interaction. To this end, we constructed cDNA fragments encoding the different truncations of Rnf111 and cGAS and expressed them in HEK293T cells. The IP results showed that Rnf111 interacted with cGAS with multiple binding domains, and the interaction between them was independent of the sumo-interacting motif (SIM) and RING domain of Rnf111 (Fig 4G). We also found the regions of aa1-120 and aa241-380 of cGAS were required for its interaction with Rnf111 (Fig 4H). Taken together, these data demonstrated that Rnf111 interacted with cGAS, and cGAS might be a substrate of Rnf111 Nedd8 E3 enzyme.

### RNF111 promotes the neddylation of cGAS

**Fig 5:**
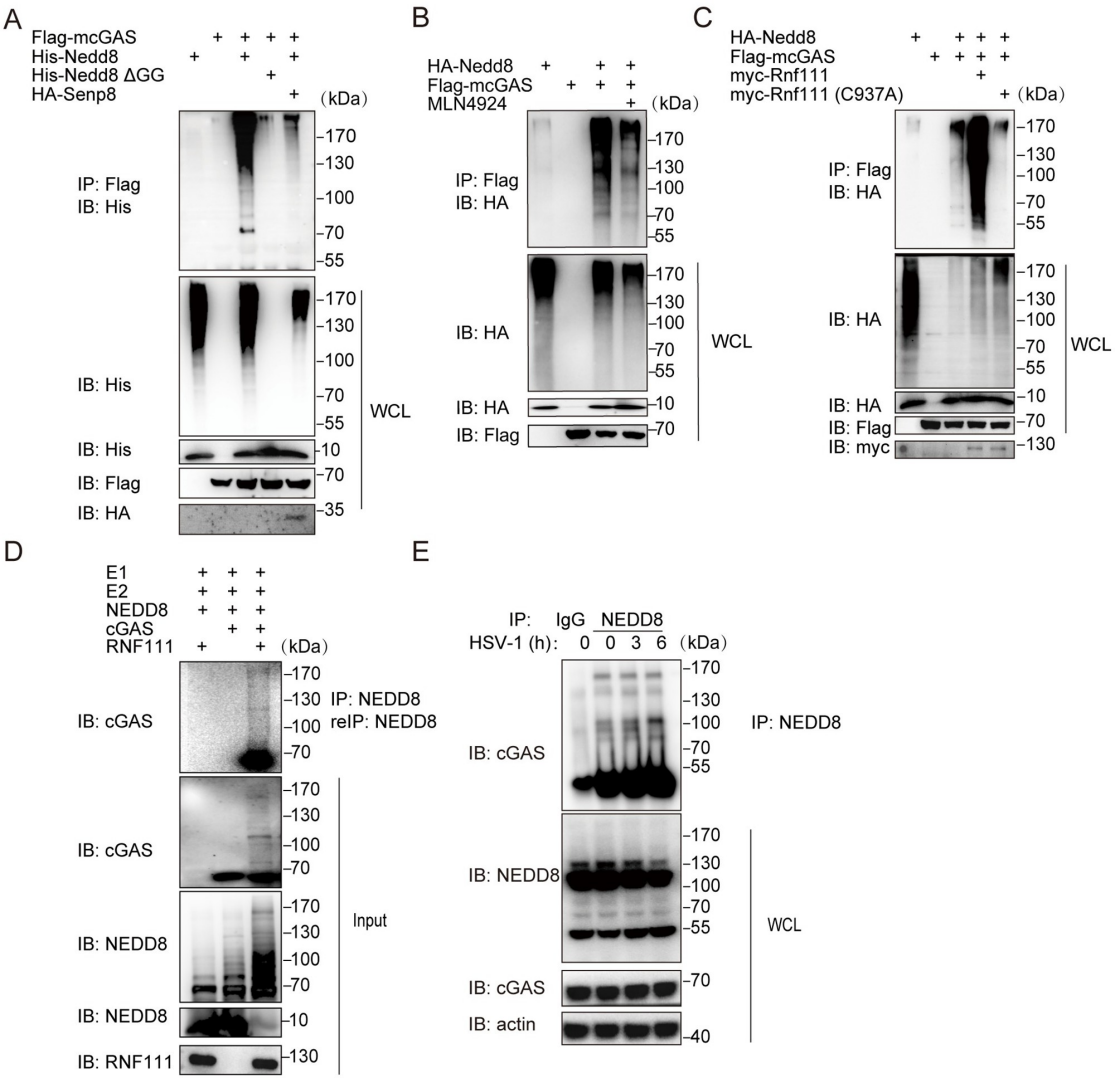
Molecular characterization of cGAS neddylation. (A) HEK293T cells were transfected with indicated plasmids. 48h after transfection, cells were denatured with 1% SDS, then cells were lysed and immunoprecipitation with anti-Flag antibody and then analyzed by immunoblotting with indicated antibodies. (B) HEK293T cells were transfected with indicated plasmids. 45h after transfection, one group of cells was treated with 5mM MLN4924 for 3h, then cells were subjected to denatured immunoprecipitation with anti-Flag antibody and then analyzed by immunoblotting with indicated antibodies. (C) HEK293T cells were transfected with HA-Nedd8, Flag-cGAS, myc-Rnf111 (WT), myc-Rnf111(C937A). 48h after transfection, cells were subjected to denatured immunoprecipitation with anti-Flag antibody and then analyzed by immunoblotting with indicated antibodies. (D) Recombinant cGAS was incubated with NEDD8, E1, UBE2M and RNF111 in the presence of ATP, after the reaction, proteins were subjected to SDS-PAGE, and immunoblotting with indicated antibodies. (E) MEFs were infected with HSV-1 (MOI = 0.3) for indicated times, then cell lysis was subjected to denatured immunoprecipitation with anti-NEDD8 antibody and then analyzed by immunoblotting with indicated antibodies.

Next, we investigated whether cGAS is subjected to neddylation modification. Exogenous Nedd8 and cGAS were transfected individually or in combination into HEK293T cells followed by denatured-immunoprecipitation assay. cGAS was poly-neddylated by wild-type Nedd8 but not Nedd8 ΔGG that is conjugation-defective due to the Gly-75/76 deletion [29] (Fig 5A). Consistent with the fact that Senp8 is a deneddylase, cotransfection of Senp8 greatly inhibited the neddylation of cGAS (Fig 5A). Furthermore, we found that MLN4924 pretreatment strongly inhibited cGAS neddylation (Fig 5B). Consistent with the Rnf111-cGAS binding, cGAS polyneddylation was significantly promoted by Rnf111, but not by its enzymatic dead mutant Rnf111-C937A (Fig 5C). Finally, the *in vitro* neddylation assay confirmed that RNF111 facilitated the polyneddylation of cGAS (Fig 5D). Endogenous denatured-immunoprecipitation also showed that cGAS was poly-neddylated, which was enhanced by HSV-1 infection (Fig 5E). Thus, RNF111 is indeed the E3 NEDD8 ligase for cGAS. To identify which lysine residue of cGAS was modified by neddylation, a NEDD8 R74K was employed to distinguish NEDD8 and ubiquitin modification sites by mass spectrometry (MS) in both human and mouse [30]. In human, eight lysine residues of cGAS were identified under NEDD8-modification: Lys21, Lys47, Lys187, Lys231, Lys292, Lys299, Lys392, and Lys421. We also found eight lysine residues of cGAS were modified by NEDD8 in mouse: Lys173, Lys205, Lys217, Lys286, Lys335, Lys382, Lys409, Lys472 (S6A Fig and S1 Table). Among them, the modifications at Lys231 and Lys421 of human cGAS were conserved to Lys217 and Lys409 of mouse cGAS, respectively.

To investigate their role in neddylation, each of the eight lysine residues of human cGAS was mutated to arginine. We found none of the single site mutations totally abolished the neddylation of cGAS, while cGAS with single mutation of K47R, K187R, K231R, K292R, K299R, K392R and K421R all lead to the reduced cGAS neddylation (S6B Fig), indicating that cGAS was modified by NEDD8 at multiple lysine residues. To further examine the role of these neddylation sites in innate immune response, cGAS carrying each of single site mutation was transfected to HEK293 cells. Intriguingly, we found mutations at each of the two conserved neddylation sites (K231R and K421R) strongly impaired the transcription of *Ifnb* and *Cxcl10* induced by HT-DNA (S6C Fig).

### Neddylation facilitates cGAS activity

**Fig 6:**
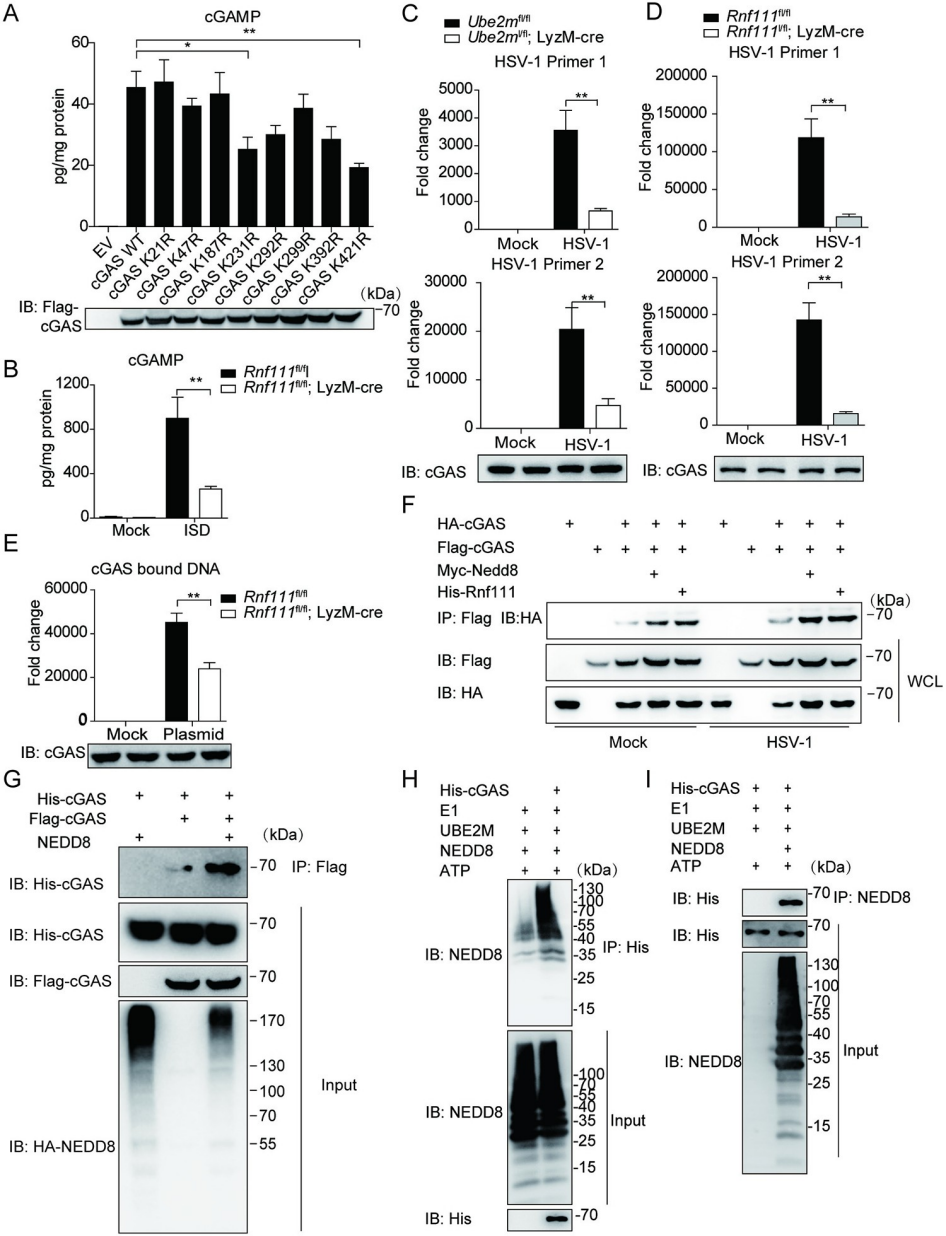
Neddylation promotes the activation of cGAS. (A) HEK293T cells were transfected with indicated plasmids. 24h after transfection, cells were stimulated with HT-DNA for 4h before cells were harvested for cGAMP detection. (B) *Rnf111* deficiency or control BMDM were transfected with ISD for 4h, cell lysates were heat denatured, and cGAMP was measured by ELISA. (C) *Ube2m* deficiency BMDMs were infected with HSV-1 (MOI = 0.5) for 3h, after adding human cDNA as an external reference, cell lysates were immunoprecipitated with anti-cGAS antibody, then cGAS-bound DNA was extracted and quantified by qRT-PCR by normalized to human GAPDH. (D) *Rnf111* deficiency BMDMs were infected with HSV-1 (MOI = 0.5) for 3h, after adding human cDNA as an external reference, cell lysates were immunoprecipitated with anti-cGAS antibody, then cGAS-bound DNA was extracted and quantified by qRT-PCR by normalized to human GAPDH. (E) *Rnf111* deficiency BMDMs were transfected with an empty plasmid for 3h, after adding human cDNA as an external reference, cell lysates were immunoprecipitated with anti-cGAS antibody, then cGAS-bound DNA was extracted and quantified by qRT-PCR by normalized to human GAPDH. (F) HEK293T cells were transfected with indicated plasmids for 45h and then infected with HSV-1 (MOI = 0.3) for 4h, the cell lysates were immunoprecipitated with an anti-Flag antibody and then immunoblotted with indicated antibodies. (G) HA-NEDD8 and Flag-cGAS were coexpressed in HEK293T cells, 48h later cells were collected and cell lysates were incubated with recombinant His-cGAS, the interaction was analyzed by immunoprecipitated with anti-Flag beads and immunoblotted with indicated antibodies. (H) Poly-NEDD8 chains were generated *in vitro*, followed by incubating with His-cGAS. After immunoprecipitated with anti-His antibody, and then immunoblotted with indicated antibodies. (I) Poly-NEDD8 chains were generated *in vitro*, followed by incubating with His-cGAS. After immunoprecipitated with anti-NEDD8 antibody, and then immunoblotted with indicated antibodies. Graphs are presented as means ± SEM, data are representative of three independent experiments, *P <0.05; **P <0.01 (One-way ANOVAs followed by Tukey’s post hoc test).

We then investigated the influence of neddylation on the activation of cGAS. Consistent with our finding that the neddylation was required for cGAS-STING activation, mutation of K231 or K421 reduced cGAMP synthesis induced by HT-DNA (Fig 6A), and the level of cGAMP was remarkably decreased in *Rnf111* deficiency BMDMs after ISD stimulation (Fig 6B). To further address whether the neddylation of cGAS affected its DNA binding ability, we used qRT-PCR to quantify the amount of HSV-1 DNA after cGAS immunoprecipitation. The results showed that overexpression of Nedd8 or Rnf111 promoted cGAS binding to HSV-1 DNA whereas the deficiency of either *Ube2m* or *Rnf111* compromised DNA binding of cGAS (Fig 6C and 6D and S7A Fig). Notably, Nedd8 ΔGG had no effects on cGAS DNA binding (S7B Fig). In addition, the input of HSV-1 was higher in *Ube2m* or *Rnf111* deficiency BMDMs when compared with their counterparts (S7C and S7D Fig). To exclude the possibility that cGAS bind to DNA indirectly through interacting with other components of HSV-1 (such as capsid), we transfected BMDM with an empty vector DNA. The result confirmed that *Rnf111* deficiency led to less DNA binding of cGAS (Fig 6E and S7E Fig). To be mentioned, either *Ube2m* or *Rnf111* deficiency increased the total DNA input quantity (S7C–S7E Fig), which is consistent with the previous observation that cGAS-STING signaling supports the clearance of cytosolic DNA and DNA viruses [31].

Upon DNA stimulation, cGAS forms a 2:2 dimer with DNA, and the dimerization of cGAS is essential for its activation [32]. We then examined the activation of cGAS by measuring cGAS dimerization status. This *in vivo* experiment showed that overexpression of either Nedd8 or Rnf111 strengthened the formation of cGAS homodimer in the presence of ISD (Fig 6F). The immunoprecipitation experiment further showed the neddylated-cGAS had a stronger affinity to form dimer with each other when Nedd8 is co-transfected (Fig 6G). It has been shown that NEDD8 acts as a nexus to promote protein interaction [33]. We found cGAS interacted with poly-NEDD8 chains *in vitro* (Fig 6H and 6I), suggesting that neddylation might enhance cGAS dimerization through NEDD8 chains as nexus. Taken together, these data indicated that neddylation facilitated the cGAS dimerization and strengthened its DNA-binding activity.

### Ube2m and Rnf111 are critical for innate immune response to HSV-1 infection *in vivo*

**Fig 7:**
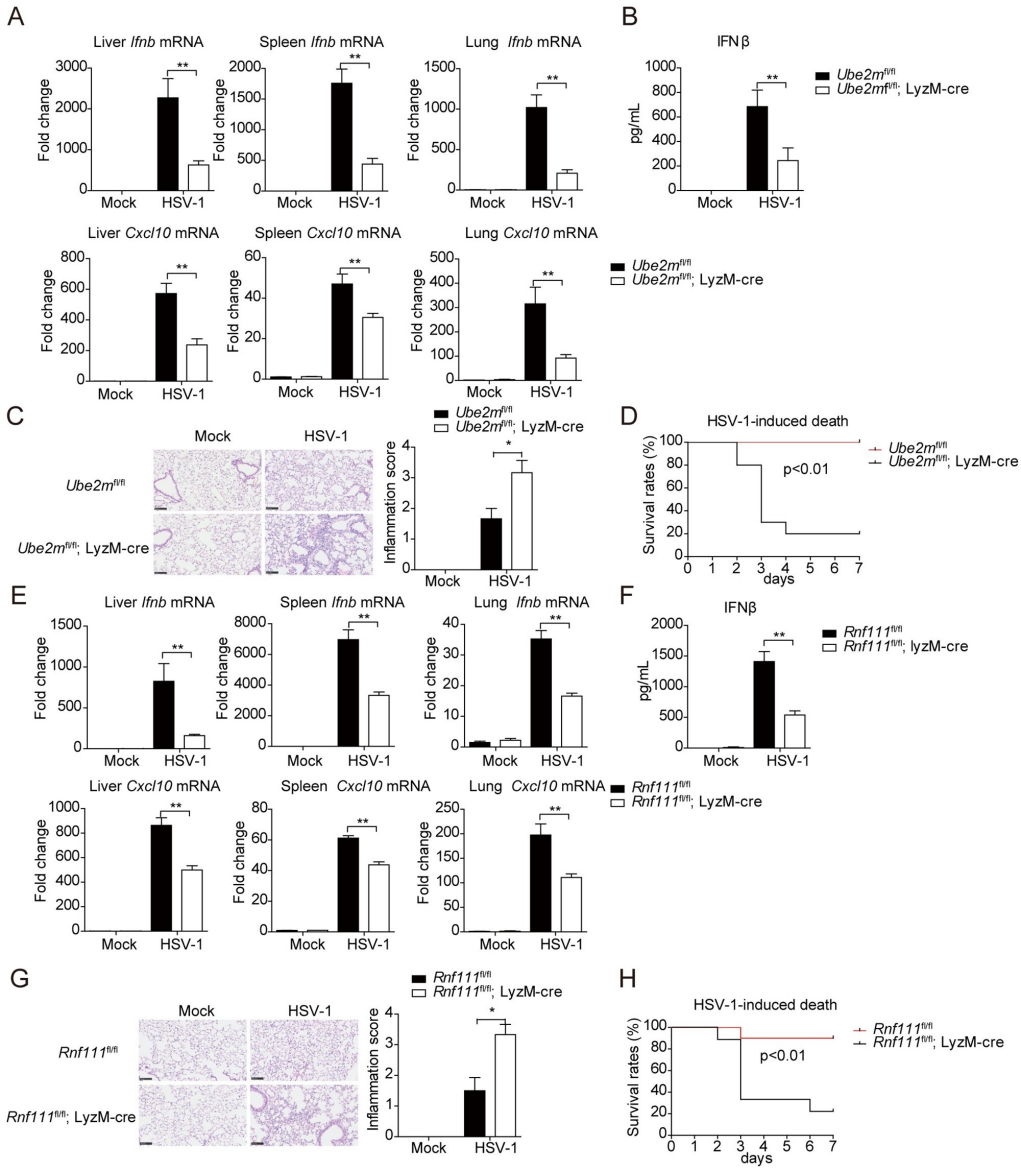
UBE2M-RNF111 mediated neddylation pathway is indispensable for the innate defense against HSV-1 infection. (A) Mice were injected intravenously with HSV-1 (1.5×10^7^ pfu per mouse) for 12h, tissues from *Ube2m* cKO or the control group were harvest and the relative expression of *Ifnb and Cxcl10* in livers, spleens and lungs were measured by qRT-PCR, respectively (n = 6–10). (B) Mice were injected intravenously with HSV-1 (1.5×10^7^ pfu per mouse) for 12h, the serum was harvested and the concentration IFNβ was analyzed with ELISA (n = 6–8). (C) Mice were injected intravenously with HSV-1 (1.5×10^7^ pfu per mouse) for 72h, lungs were harvested and inflammatory cells infiltration were analyzed by H&E staining (left), scale bars represent 100 μm. The presence of inflammation was scored and counted (right, n = 5). (D) Mice of the *Ube2m* cKO or the control groups were injected intravenously with HSV-1 (6×10^7^ pfu per mouse), and the survival rates were monitored for 7 days (n = 10). (E) Mice were injected intravenously with HSV-1 (1.5×10^7^ pfu per mouse) for 12h, tissues from *Rnf111* cKO or the control group was harvest, and the relative expression of *Ifnb* and *Cxcl10* in livers, spleens and lungs were measured by qRT-PCR (n = 6–10). (F) Mice were injected intravenously with HSV-1 (1.5×10^7^ pfu per mouse) for 12h, the serum was harvested and the concentration IFNβ was measured with ELISA (n = 6–10). (G) Mice were injected intravenously with HSV-1 (1.5×10^7^ pfu per mouse) for 72h, lungs were harvested and inflammatory cells infiltration were analyzed by H&E staining (left), scale bars represent 100 μm. The presence of inflammation was scored and counted (right, n = 5). (H) Mice of the *Rnf111* cKO or the control groups were injected intravenously with HSV-1 (6×10^7^ pfu per mouse), and the survival rates were monitored for 7 days (n = 10). Graphs are presented as means ± SEM, data are representative of three independent experiments, *P <0.05; **P <0.01 (Mann-Whitney test for C and G, Mantel-Cox test for D and H, One-way ANOVAs followed by Tukey’s post hoc test for the rest).

To further investigate the *in vivo* antiviral function of UBE2M-RNF111 mediated neddylation modification of cGAS, *Ube2m* deficient mice were infected with HSV-1. Compared with control littermates, the expression of *Ifnb* and *Cxcl10* were markedly decreased in the liver, spleen and lung of *Ube2m* deficient mice (Fig 7A). Moreover, the serum level of IFNβ was also remarkably decreased in *Ube2m* deficient mice compared with control littermates (Fig 7B). In addition, we observed more inflammatory cells infiltrated to the lungs of *Ube2m* deficient mice after HSV-1 infection (Fig 7C). Consistently, *Ube2m* deficient mice exhibited much lower survival rate after HSV-1 infection compared with control mice over a period of 7 days (Fig 7D). In contrast, *Ube2f* deficiency influenced neither the transcription of *Ifnb* and *Cxcl10* genes nor the mouse survival after HSV-1 infection, as compared with control mice (S8A and S8B Fig).

Finally, the effect of *Rnf111* deficiency was examined *in vivo*. Compared with control mice, *Rnf111*^fl/fl^; LyzM-cre mice displayed severe defects in expression of *Ifnb* and *Cxcl10* in liver, spleen and lung after HSV-1 infection (Fig 7E). Moreover, the serum level of IFNβ was remarkably decreased in *Rnf111* deficient mice compared with the control group (Fig 7F). The *Rnf111* deficient mice also exhibited significantly higher infiltration of inflammatory cells in the lung and much lower survival rate compared with the control group after HSV-1 infection (Fig 7G and 7H). Collectively, *Ube2m* or *Rnf111* deficiency mice exhibited severe defects in innate immune response and were susceptible to HSV-1 infection.

## Discussion

The neddylation pathway has been shown to play an important role in inflammatory response [14,15,34–36]. It has also been demonstrated that pathogens such as *Listeria monocytogenes* can inhibit neddylation to avoid the immune response, which implies that neddylation may play critical roles in antipathogenic immunity [16,37–39]. The host cells can sense DNA released from bacteria to the phagosomes or the cytosol and elicit a series of immune responses [40]. The cGAS-STING pathway plays critical roles in antipathogenic immunity by sensing cytosolic DNA. Notably, cGAS or STING deficiency mice are unable to produce type I interferon and susceptible to *Listeria monocytogenes* [41]. Whether and how neddylation pathway modulates the cGAS/STING signals is previously unknown.

In this study, we demonstrated that the Ube2m-Rnf111 neddylation axis mediated cGAS neddylation to promote its dimerization and DNA binding affinity. *Ube2m* or *Rnf111* deficiency impaired the innate immune response to dsDNA stimulation, while *Senp8* deficiency enhanced the response. Furthermore, mice with myeloid deletion of *Ube2m* and *Rnf111*, but not *Ube2f*, were susceptible to HSV-1 infection, as evidenced by the defective in cGAS-mediated induction of type I interferons and much lower survival rates.

In mammalian cells, while only two neddylation E2s (UBE2M and UBE2F), there are little over 10 neddylation E3 [21,23]. RNF111, one of neddylation E3 has previously been linked to immunity by regulating TGF-β pathway [42], our investigation demonstrated that DNA stimulation increased the protein levels of Rnf111, which might explain the enhanced interaction between Rnf111 and cGAS upon DNA stimulation. *Rnf111* deficiency impaired DNA elicited induction of cGAMP but not cGAMP-induced transcription of downstream genes, while reconstitution of *Rnf111* but not the E3-defective mutant *Rnf111* C937A could rescue DNA-induced downstream genes, consistent with the result that overexpression of WT but not C937A *Rnf111* promoted the neddylation of cGAS, indicating that the enzyme activity of RNF111 is essential for activation of the cGAS-STING pathway. RNF111 acts as E3 for both ubiquitin and NEDD8, with UBE2N and UBE2d2a as the E2 for ubiquitylation [43,44], and with UBE2M as the E2 for neddylation [45]. *Ube2m* but not *Ube2n* or *Ube2d2a* deficiency impaired DNA-triggered innate immune response, indicating that Rnf111 acted as a NEDD8-specific ligase to promote cGAS the activation.

In this study, we found *Ube2m* or *Rnf111* deficiency impaired the DNA binding ability of cGAS, as well as the subsequent signaling events, including phosphorylation of TBK1, IRF3, p65, and IκBα, suggesting that UBE2M and RNF111 mainly involved in the regulation of DNA triggered innate immune responses through the cGAS-STING signaling. It should be noted that the E1 of neddylation pathway has been reported to promote HSV-1 induced type I interferon by facilitating NF-κB nuclear translocation without affecting IRF3 [46]. These observations indicated that the mechanisms underlying the regulation of neddylation on DNA triggered innate immune responses might be rather complex, and might be influenced by the cell types being tested, the types of stimulation employed, and other potential factors related to experimental setup differences. On the other hand, these observations also suggested that different components of neddylation pathway may facilitate antiviral function in different manners through neddylation pathway alone and/or by cross-talking the other unknown signaling pathways. The exact functions and mechanisms of how different components of neddylation pathway affect DNA triggered innate immunity still required further investigation.

In addition, we found that knocking out *Ube2m* or *Rnf111* upregulated SeV or Poly(I:C) induced production of type I IFNs, which was consistent with the previous observation that Rnf111 functions to impair the antiviral immunity to RNA virus [47]. These results suggested that Ube2m-Rnf111 axis specifically facilitated the DNA triggered anti-viral activity through the cGAS-STING pathway. How Ube2m or Rnf111 attenuated the RNA sensing pathway is unknown and it is interesting to investigate the underlying mechanisms in the future.

It has been reported that cGAS is on a balance of monomer and dimer, dsDNA may shift the balance to dimeric status, and factors promote the dimeric status may increase the sensitivity of cGAS to dsDNA [32,48–50]. Moreover, TRIM56-mediated ubiquitination facilities cGAS activation by promoting its dimerization and potentiating the activation of cGAS [51]. Here we demonstrated that Nedd8 and Rnf111 promoted the dimerization of cGAS. In recent years, several NEDD8-interacting proteins have been identified, and NEDD8 acts as a nexus to link two proteins, thus we speculate that NEDD8 links the cGAS to strengthen the dimer [23,52,53]. Our *in vitro* assay showed that compared to non-neddylated cGAS, polyneddylated cGAS facilitated the formation of homodimer much better. Indeed, cGAS interacts with poly-NEDD8 chains. Consistent with previous observation that cGAS dimerization supports DNA sensing and the subsequent activation of cGAS [32,54], we showed that Nedd8 and Rnf111 facilitate cGAS DNA binding, while the conjugation-defective mutant Nedd8 failed to do so, and *Ube2m* or *Rnf111* deficiency impairs the DNA-binding ability of cGAS and cGAS activation.

It is well established that DNA damage gives rise to micronuclei, which activates cGAS and subsequent proinflammatory response [55]. Meanwhile, neddylation has been demonstrated to play critical roles in regulating the cellular response to DNA damage, and RNF111 has been shown to cooperate with RNF168 to facilitate the DNA damage repair process in nucleus [23,45,56]. In this study, we demonstrated that Rnf111 cooperates with cGAS to detect DNA, which is independent of Rnf168. Thus, we speculate that Rnf111 may have a role in adjusting the balance of DNA repair and DNA damage-induced immune response, which is an interesting topic for future investigation.

**Fig 8:**
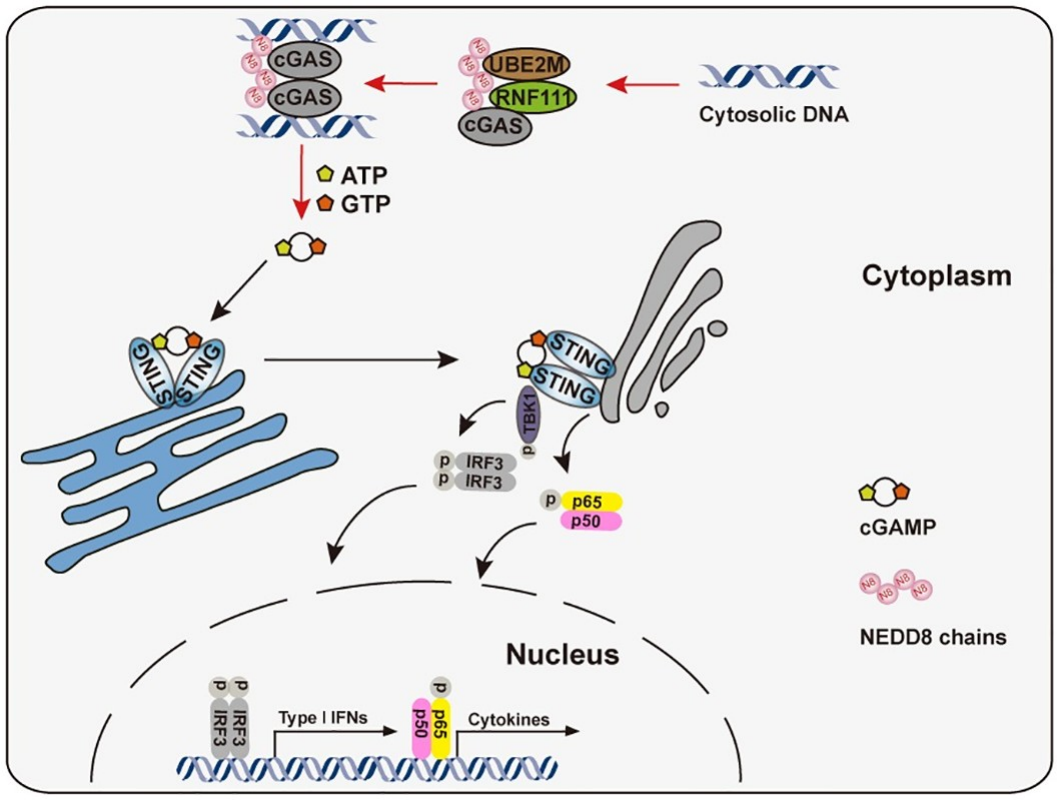
Models of RNF111-facilitated neddylation potentiated the activation of cGAS. In the presence of cytosolic DNA, the Ube2m-Rnf111 neddylation axis facilitated the neddylation of cGAS, which in turn promoted the dimerization and enhanced the DNA-binding ability of it. In the end, the Ube2m-Rnf111 neddylation axis potentiated the cGAS-STING antiviral signaling.

In summary, we defined that Rnf111-mediated neddylation positively modulates the cGAS-mediated DNA sensing immune pathway (Fig 8). It is generally accepted that the cGAS-STING pathway suppresses the development of cancer [57]. RNF111 was considered to have tumor suppressive activity [58–60], our study implied that it might partially be attributable to its neddylation modification of cGAS to facilitate immune responses. Previous studies indicate the neddylation inhibitor MLN4924 exhibit promising antitumor effects [61–63], in light of MLN4924 treatment mediated inhibition of cGAS pathway in this study, the immune-repressive side effect of MLN4924 should be taken into consideration in the future.

## Materials and methods

### Ethics statement

All the animals were treated according to the Guide for Care and Use of Laboratory Animals, approved by the Animal Experimentation Ethics Committee of China Pharmaceutical University (Approval number: 2021-02-002).

### Animals

The *Ube2m*^*fl/fl*^ mice and *Ube2f*^*fl/fl*^ mice were generated as described [22]. The *Rnf168*^*−/−*^ mice on the C57BL/6 background were gifted from Daming Gao (Chinese Academy of Sciences) [28]. The LyzM-cre mice were gifted from Guangxun Meng (Chinese Academy of Sciences). *Rnf111*^*fl/fl*^ mice on the C57BL/6 background were purchased from Nanjing Biomedical Research Institute of Nanjing University via Cas9 technology with exons 5–6 floxed. The mice were bred in specific pathogen-free facilities at Center for new drug safety evaluation and research, China Pharmaceutical University.

### Plasmids

NEDD8, cGAS, STING, TBK1, IRF3, SENP8, RNF111 cDNAs were obtained using PCR amplified and cloned into mammalian expression vectors as indicated.

### Cell culture and transfection

MEF, HEK293T cells were maintained in Dulbecco’s modified Eagle’s medium supplemented with 10% fetal bovine serum. L929 cells were maintained in RPMI1640 medium supplemented with 10% fetal bovine serum. BMDMs (bone marrow-derived macrophages) were prepared as described previously [64]. Cells were grown in a humidified atmosphere with 5% CO2 at 37°C. Lipofectamine 2000 (Invitrogen) was used for transfection of HT-DNA or ISD according to the manufacturer’s instructions. Lipofectamine RNAiMAX Transfection Reagent (Thermo Fisher) was used for transfection of siRNA, and X-GENE HP from Roche (06366 236 001) was used for transfection of plasmids into BMDMs.

### Antibodies and reagents

HSV-1 (F strain) were kindly provided by Dr. Wentao Qiao (Nankai University), SeV (strain Cantell) were kindly provided by Dr. Chunfu Zheng (Suzhou University). The anti-RNF111 antibody (PA5-58449) was purchased from Thermo Fisher, the anti-RNF111 antibody (TA315537) was purchased from OriGene, the anti-UBE2M antibody (4913S) was purchased from Cell Signaling Technology, the anti-UBE2F antibody (sc-398668) antibody was purchased from Santa Cruz, the anti-cGAS antibodies were purchased from Santa Cruz (sc-515777) and Cell Signaling Technology (31659S), the anti-TBK1 antibody was from Abcam (ab40676). The anti-IRF3 (D83B9), anti-Phospho-IRF3 (4D4G), anti-Phospho-STING (85735), and anti-Phospho-TBK1 (D52C2), anti-Phospho-NF-κB p65 (93H1), anti-NF-κB p65 (D14E12), anti-Phospho-IκBα (14D4) and anti-IκBα (L35A5) antibodies were from Cell Signaling Technology. Anti-β-Actin, anti-Flag antibodies were from Sigma, anti-HA, anti-myc and anti-His antibodies were from Santa Cruz.

TnT Quick Coupled Transcription/Translation System was from Promega (L1170). Duolink In Situ Detection Reagents Red (DUO92008), Duolink In Situ PLA Probe Anti-Rabbit PLU (DUO92002) and Duolink In Situ PLA Probe Anti-Mouse MINUS (DUO92001) were from Sigma.

cGAMP was from Invivogen and was delivered into cultured cells by the digitonin permeabilization method as previously described [64]. HT-DNA was from Sigma. Interferon stimulatory DNA (ISD) was prepared by annealing equimolar amounts of sense and antisense DNA oligonucleotides at 95°C for 10 minutes before cooling to room temperature. Oligonucleotides used as follows:

ISD (sense), 5’-TAC AGA TCT ACT AGT GAT CTA TGA CTG ATC TGT ACA TGATCT ACA-3’;
ISD (antisense), 5’-TGT AGA TCA TGT ACA GAT CAG TCA TAG ATC ACT AGT AGA TCT GTA-3’.

### RNA interference

The siRNAs duplexes were synthesized from Gene-Pharma. The sequences of siRNAs are shown as follows:

UBA3 siRNA for mouse: 5’-GGUCGCUGGAACCAUGUAATT-3’;
RNF111 siRNA1 for mouse: 5’- GCAGAAGUGGAGAUGAUUATT-3’;
RNF111 siRNA2 for mouse: 5’- GCAGAAGUUGUGGACCUUATT-3’;
SENP8 siRNA for mouse: 5’- GCAAUCAGAUGUCUCACUATT-3’;
UBE2d2a siRNA for mouse: 5’- CAAUCCAGAUGAUCCUUUATT-3’;
UBE2n siRNA for mouse: 5’- GCACCUAAAGUACGUUUCATT-3’;
RBX1 siRNA for mouse: 5’- GAGAGUGGGAGUUCCAGAATT -3’;
RBX2 siRNA for mouse: 5’- GCAAGAGGACUGUGUUGUGTT -3’;
CBL siRNA for mouse: 5’- CCUCAUGAGUCAGGGCUAUTT -3’;
DCN1 siRNA for mouse: 5’- GCAUCAGUGUGUUGAUCAUTT -3’;
RNF111 siRNA1 for human 5’- GCAGGAGUUGAGAUGAUUATT -3’;
RNF111 siRNA2 for human: 5’- GGAAGAAACGAGAAGUGUUTT-3’.

### Real-time PCR

The samples were homogenized in TRIzol reagent (Invitrogen). Total RNA was extracted according to the manufacturer’s suggested protocol. The RNA was converted to cDNA using the HiScript 1st Strand cDNA Synthesis Kit (Vazyme). Quantitative PCR was performed using FastStart Universal SYBR GREEN MASTER MIX (Roche). The cycle time values were normalized to GAPDH of the same sample. The data were normalized to the control group, with the control group as 1. PCR primers of indicated target genes are shown in S2 Table.

### Cytokine enzyme-linked immune sorbent assays (ELISA)

Concentrations of cytokines in mouse serum were measured by Veri-Kine Kit (PBL Assay Science) according to the manufacturer’ s instructions.

### Immunoprecipitation and immunoblot analysis

For non-denature immunoprecipitation, cells were lysed with 0.2% Triton buffer (50 mM Tris HCl pH 7.4, 1 mM EDTA, 150 mM NaCl, 20mM N-Ethylmaleimide, 0.2% Triton X-100) supplemented with a protease inhibitor cocktail (Sigma), followed by centrifugation at 12,000g for 15 min at 4°C. The supernatants were incubated with anti-Flag M2 affinity beads or indicated antibodies followed by protein A/G agarose beads.

For denaturing immunoprecipitation, cells were denatured in 1% SDS buffer (50 mM Tris-HCl, pH 7.5, 150 mM NaCl, 1% SDS, 10 mM DTT) by heating for 30 min. The buffer was then diluted 10 times with Lysis buffer (50mM Tris-HCl pH 7.5, 150 mM NaCl, 1 mM EDTA, 1% Triton X-100), followed by centrifugation at 12,000g for 15 min at 4°C. The supernatants were incubated with anti-Flag M2 affinity beads or indicated antibodies followed by protein A/G agarose beads.

Cell lysates or immunoprecipitates were separated by SDS–PAGE and then transferred to PVDF membrane (Millipore). The membranes were then incubated with appropriate primary and secondary antibodies, and then visualized with the Bio-Rad system (Bio-Rad, Germany).

To collect cytosol and nuclear fraction separately, a nuclear and cytoplasmic protein extraction kit (Sangon Biotech) was employed, and practiced according to the manufacturer’ s instructions.

### Native PAGE assay

For IRF3 dimerization assay, cells were lysed with 0.2% triton buffer (50 mM Tris HCl pH 7.4, 1 mM EDTA, 150 mM NaCl, 20mM N-Ethylmaleimide, 0.2% Triton X-100, 1mM NaF, 1mM NaVO3) supplemented with a protease inhibitor cocktail (Sigma), the following procedure was carried out as described previously [65].

### Immunofluorescence and confocal microscopy

Cells seeded onto glass coverslips were fixed for 30min with 4% paraformaldehyde in PBS and permeabilized with 0.3% Triton X-100 in PBS for 15 minutes, before blocking with 5% BSA in PBS for 1 h. Then, cells were stained with indicated primary antibodies overnight at 4°C. After washing with PBS for 3 times, the cultures were incubated with AlexaFluor 488 conjugated anti-Rabbit and Cyanin3 conjugated anti-mouse secondary antibodies for 1 hour. The nuclei were stained with DAPI for 90 seconds and then washed with PBS for 3 times.

### Proximity ligation assay (PLA)

PLA was performed as per manufacturer’s instructions (Sigma). Briefly, MEF or L929 cells seeded onto glass coverslips were fixed for 30min with 4% paraformaldehyde in PBS and permeabilized with 0.3% Triton X-100 in PBS for 15 minutes, before blocking with Duolink Blocking Solution for 1h at 37°C. Then cells were incubated with primary antibodies overnight at 4°C. After washing with buffer A for 3 times, the cultures were incubated with PLA probes (DUO92001, DUO92002) for 1 h at 37°C. After washing with buffer A for another 3 times, the cultures were incubated with Ligase diluted in Ligation buffer for 30 min at 37°C. After washing with buffer A for 3 times, the cultures were incubated with Polymerase diluted in Amplification buffer for 100 min at 37°C. After washing with 1×Buffer B for 3 times followed by 0.01×Buffer B once, the cultures were mounted with Duolink In Situ Mounting Medium with DAPI.

### Recombinant proteins

6×His-tagged human cGAS constructs were cloned into the pET-28a (+) vector. The plasmids were transformed into the Rosetta (DE3) E. Coli strain. Expression of the cGAS-fusion proteins was induced with 0.1 mM IPTG at 17°C for 24 h. Bacterium were lysed in Lysis Buffer (50 mM Tris-HCl pH 7.4, 300 mM NaCl, 1% Triton X-100, 20 mM imidazole, 10 mM β-ME, 1 mM PMSF). After centrifugation, the supernatants were collected and incubated with 20 μL Ni-NTA agarose beads (Qiagen) for 2 h at 4°C. After extensively washing with Lysis Buffer containing 20 mM imidazole, the precipitates were eluted with TBS containing 300 mM imidazole. cGAS proteins were frozen and stored in −80°C with exchange buffer (20 mM Tris-HCl pH 7.5, 100 mM NaCl, 0.5mM DTT, 0.2mM PMSF).

### *In vitro* NEDD8 chain formation

The procedure was carried out as described previously [20]. Briefly, poly-NEDD8 was produced *in vitro* with 0.15 μM NAE, 10 μM UBE2M and 20 μM NEDD8 in reaction buffer (50 mM Tris–HCl pH 8.0, 200 mM NaCl, 20% glycerol, 10 mM MgCl2, 10 mM ATP, 0.6 mM DTT) for 4 h at 30°C.

### *In vitro* neddylation and protein binding

His-tag Rnf111 was generated by the TNT Quick Coupled in vitro transcription/translation kit (Promega). *In Vitro* neddylation was performed in in the reaction buffer (50mM Tris-HCl pH 7.5, 1 mM DTT, 2 mM NaF, 10 mM MgCl_2_, 5 mM ATP) for 1 h at 37°C. In a 50μl reaction system, 10μl *in vitro* translated protein, 1μg NEDD8, 200 ng NEDD8 E1 and 400 ng UBE2M were added. The samples were incubated with anti-NEDD8 antibody followed by protein A/G agarose beads, then denatured in 1% SDS buffer (50 mM Tris-HCl, pH 7.5, 150 mM NaCl, 1% SDS, 10 mM DTT) by heating for 5 min. The buffer was then diluted 10 times with reaction buffer, and re-IP with anti-NEDD8 antibody and protein A/G agarose beads, followed with immunoblot analysis.

### cGAMP extraction and quantification

Cells were stimulated with ISD for 4h, the cells were then harvested and lysed with M-PER Mammalian Protein Extraction Reagent (Thermo Fisher). After centrifugation at 14,000g for 10 min, the protein level of supernatant was measured with BCA kit (Beyotime Biotechnology). Then the left supernatant was heated at 95°C for 5min and centrifuged at 14,000g for 10 min, the final supernatant was taken to measure the concentration of cGAMP with a cGAMP ELISA kit (Cayman Chemical).

### Histological assessments of lungs

At 4 days after HSV-1 infection, lungs were fixed in paraffin, cut into sections, stained with H&E solution and images were captured with a Nanozoomer slide scanner (Nanozoomer 2.0-RS). The degree of inflammation was assessed using a 5-point scale [66]: 0, no observable inflammation; 1–4 represents mild, moderate, marked, or severe inflammation, respectively.

### Mass spectrometry

Ubiquitin, as well as NEDD8, were covalently bond to the lysine of the substrates with their C-terminal RGG residue. To distinguish Ubiquitin and NEDD8, we employed a NEDD8 with R74K mutation. The R74K-NEDD8-modified substrates can be digested by Lys-C to expose the KGG residue for mass spectrometry. HEK293T cells were transfected with FLAG-cGAS and His-NEDD8 (R74K). 48 hours after transfection, cells were harvested and denatured-IP was operated as mentioned above. After separated by SDS-PAGE and stained with trypan blue, protein bands corresponding to cGAS were collected and analyzed by mass spectrometry. The mass spectrometry proteomics data have been deposited to the ProteomeXchange Consortium via the PRIDE [67] partner repository with the dataset identifier PXD024088.

### Statistical analysis

Results were presented as means ± SEM. For statistical analysis, GraphPad Prism Software 6 (La Jolla, CA, USA) was used. One-way ANOVAs followed by Tukey’s post hoc test were utilized for multiple group comparisons of the parameters. The Differences in the mouse survival curves were analyzed with the Mantel-Cox test. Differences in the frequencies of histology scores were analyzed by the Mann-Whitney test. All tests were considered statistically significant at *P* < 0.05.

## Supporting information

S1 Fig Neddylation positively regulates dsDNA-triggered signaling. (A) MEFs were transfected with negative control (N.C.) or *Uba3* siRNAs for 48h, then stimulated with HSV-1 (MOI = 1) or ISD for 6h, the transcription of *Ifnb*, *Ifna4* and *Cxcl10* were measured by qRT-PCR. (B) L929 were transfected with N.C. or *Uba3* siRNAs for 48h, then stimulated with HT-DNA for 6h, the transcription of *Ifnb*, *Ifna4* and *Cxcl10* were measured by qRT-PCR. (C) MEFs or L929 were transfected with the indicated siRNA, and the *Uba3* or *Senp8* mRNA was measured by qRT-PCR. (D and E) MEFs and L929 were transfected with N.C. or *Senp8* siRNAs for 48h, and then stimulated with ISD or HT-DNA for 6h, the transcription of *Ifnb*, *Ifna4* and *Cxcl10* were measured by qRT-PCR, respectively. Graphs are presented as means ± SEM, data are representative of three independent experiments, *P <0.05; **P <0.01 (One-way ANOVAs followed by Tukey’s post hoc test).
(TIF)

S2 Fig UBE2M regulates dsRNA-triggered signaling and NF-κB pathway. (A) Effects of *Ube2m* deficiency on the expression of *Ifnb* and *Ifna4* after Poly(I:C) stimulation for indicated hours in BMDM. (B) Effects of *Ube2m* deficiency on the expression of *Ifnb* and *Ifna4* after SeV (MOI = 1) infection in BMDM. (C) Effects of *Ube2m* deficiency on the phosphorylation of p65 and IκBα after ISD stimulation for the indicated time. Graphs are presented as means ± SEM, data are representative of three independent experiments, *P <0.05; **P <0.01 (One-way ANOVAs followed by Tukey’s post hoc test).
(TIF)

S3 Fig RNF111 positively regulates dsDNA-triggered signaling. (A) MEFs were transfected with indicated siRNAs for 48h, then stimulated with HT-DNA for 6h, the transcription of *Ifnb* was measured by qRT-PCR. (B) MEFs were treated with ISD or HSV-1 (MOI = 1) for indicated time, the transcription of *Rnf111* was measured by qRT-PCR. MEFs (C), L929 (D) were transfected with N.C. or *Rnf111* siRNAs for 48h, and then stimulated with ISD or HT-DNA for 6h, the transcription of *Ifnb*, *Ifna4* and *Cxcl10* were measured by qRT-PCR. (E) HFF were transfected with N.C. or *RNF111* siRNAs for 48h, and then stimulated with HT-DNA for 6h, the transcription of *IFNB*, *IFNA4* and *CXCL10* were measured by qRT-PCR. (F) MEFs were transfected with N.C. or *Rnf111* siRNAs for 48h, and then stimulated with ISD for 6h, the phosphorylation of TBK1/IRF3 were analyzed by SDS-PAGE. (G) MEFs (left), L929 (middle) or HFF (right) were transfected with the indicated siRNA, and the *RNF111* mRNA was measured by qRT-PCR. (H) MEFs were transfected with N.C. or *Rnf111* siRNAs for 48h, and then transfected with CpG-DNA for 6h, the transcription of *Ifnb*, *Ifna4* and *Il6* were measured by qRT-PCR. (I) MEFs were transfected with N.C. or *Rnf111* siRNAs for 48h, and then stimulated with cGAMP or c-di-GMP for 6h, the transcription of *Ifnb*, *Ifna4* and *Cxcl10* were measured by qRT-PCR. Graphs are presented as means ± SEM, data are representative of three independent experiments, *P <0.05; **P <0.01 (One-way ANOVAs followed by Tukey’s post hoc test).
(TIF)

S4 Fig RNF111 regulates dsRNA-triggered signaling and NF-κB pathway. (A) BMDM were harvested from *Rnf111*^fl/fl^ or *Rnf111*^fl/fl^; LyzM-cre mice respectively, the protein level of Rnf111 was confirmed by Western Blot. (B) Effect of *Rnf111* deficiency on the protein levels of cGAS and STING after ISD stimulation for indicated time. (C) Effect of *Rnf111* deficiency on the protein level of cGAS in the cytoplasmic and nuclear fractions. (D) Effects of *Rnf111* deficiency on the expression of *Ifnb* and *Ifna4* after Poly(I:C) stimulation for indicated hours in BMDM. (E) Effects of *Rnf111* deficiency on the expression of *Ifnb* and *Ifna4* after SeV (MOI = 1) stimulation in BMDM. (F) Effects of *Rnf111* deficiency on the phosphorylation of p65 and IκBα after ISD transfection for the indicated time. (G) MEFs were transfected with N.C., *Ube2n* or *Ube2d2a* siRNAs for 48h, and then stimulated with ISD for 6h respectively, the transcription of *Ifnb*, *Ifna4* and *Cxcl10* were measured by qRT-PCR. Graphs are presented as means ± SEM, data are representative of three independent experiments, *P <0.05; **P <0.01 (One-way ANOVAs followed by Tukey’s post hoc test).
(TIF)

S5 Fig Rnf168 did not influence cGAS-STING pathway. (A) Effect of *Rnf168* deficiency on the expression of *Ifnb*, *Ifna4* and *Cxcl10* after HT-DNA or ISD stimulation in BMDM. (B) Effects of *Rnf168* deficiency on phosphorylation of TBK1 after ISD or HT-DNA stimulation for indicated time. Graphs are presented as means ± SEM, data are representative of three independent experiments, *P <0.05; **P <0.01 (One-way ANOVAs followed by Tukey’s post hoc test).
(TIF)

S6 Fig Identification of cGAS neddylation sites. (A) Summary of the identified neddylation sites of cGAS using mass spectrometry data in human and mouse (FDR<0.01). The neddylation modified Lys residues were marked in red, and the conserved Lys residues under neddylation in both human and mouse cGAS were shown in bold and larger font. (B) HEK293T cells were transfected with Flag-cGAS or its K-to-R mutations. At 48h after transfection, cells were subjected to denatured immunoprecipitation with anti-Flag antibody and then analyzed by immunoblotting with indicated antibodies. (C) HEK293 cells were transfected with Flag-cGAS or its K-to-R mutations. At 24h after transfection, cells were stimulated with HT-DNA for 6h, the transcription of *Ifnb* and *Cxcl10* were measured by qRT-PCR. Graphs are presented as means ± SEM, data are representative of at least three experiments, *P <0.05; **P <0.01 (One-way ANOVAs followed by Tukey’s post hoc test).
(TIF)

S7 Fig Neddylation increases the DNA binding ability of cGAS. (A) HEK293T cells were transfected with Flag-cGAS, HA-Nedd8 and His-Rnf111, 45h after transfection, cells were stimulated with HSV-1 (MOI = 0.5) for 3h. After adding mouse cDNA as an external reference, cell lysates were immunoprecipitated with anti-Flag antibody, then cGAS-bound DNA was extracted and quantified by qRT-PCR by normalized to mouse GAPDH. (B) HEK293T cells were transfected with Flag-cGAS, HA-Nedd8 and HA-Nedd8ΔGG, 45h after transfection, cells were stimulated with HSV-1 (MOI = 0.5) for 3h. After adding mouse cDNA as an external reference, cell lysates were immunoprecipitated with anti-Flag antibody, then cGAS-bound DNA was extracted and quantified by qRT-PCR by normalized to mouse GAPDH. (C) *Ube2m* deficiency BMDMs were infected with HSV-1 (MOI = 0.5) for 3h, cells were harvested, then genomic DNA was extracted and the relative copies of HSV-1 were quantified by qRT-PCR by normalized to genomic GAPDH. (D) *Rnf111* deficiency BMDMs were infected with HSV-1 (MOI = 0.5) for 3h, cells were harvested, then genomic DNA was extracted and the relative copies of HSV-1 were quantified by qRT-PCR by normalized to genomic GAPDH. (E) *Rnf111* deficiency BMDMs were transfected with an empty plasmid for 3h, cells were harvested, then genomic DNA was extracted and the relative copies of plasmid were quantified by qRT-PCR by normalized to genomic GAPDH. Graphs are presented as means ± SEM, data are representative of three independent experiments, *P <0.05; **P <0.01 (One-way ANOVAs followed by Tukey’s post hoc test).
(TIF)

S8 Fig Ube2f does not influence anti-HSV-1 immunity. (A) Mice were injected intravenously with HSV-1 (1.5×10^7^ pfu per mouse) for 12h, tissues form *Ube2f* cKO or the control group were harvest and the relative expression of *Ifnb* and *Cxcl10* in livers, spleens and lungs were measured by qRT-PCR, respectively (n = 6–10). (B) Mice of the *Ube2f* cKO or the control group were injected intravenously with HSV-1 (6×10^7^ pfu per mouse), and the survival rates were monitored for 7 days (n = 10). Graphs are presented as means ± SEM, data are representative of three independent experiments, *P <0.05; **P <0.01 (One-way ANOVAs followed by Tukey’s post hoc test for A, Mantel-Cox test for B).
(TIF)

S1 Table Mass spectrometry data for cGAS neddylation. (XLSX)

S2 Table Sequences of primers used in qRT-PCR assay. (DOCX)
