# Correction: RNF111-facilitated neddylation potentiates cGAS-mediated antiviral innate immune response

**DOI:** 10.1371/journal.ppat.1010048

**Published:** 2021-11-03

**Authors:** Chenhui Li, Lele Zhang, Dong Qian, Mingxing Cheng, Haiyang Hu, Ze Hong, Ye Cui, Huansha Yu, Quanyi Wang, Juanjuan Zhu, Wei Meng, Jin-fu Xu, Yi Sun, Peng Zhang, Chen Wang

In Fig 4A, the DAPI-ISD stimulation image was inadvertently included twice, in place of the mock control. Please see the correct Fig 4 below.

**Fig 4:**
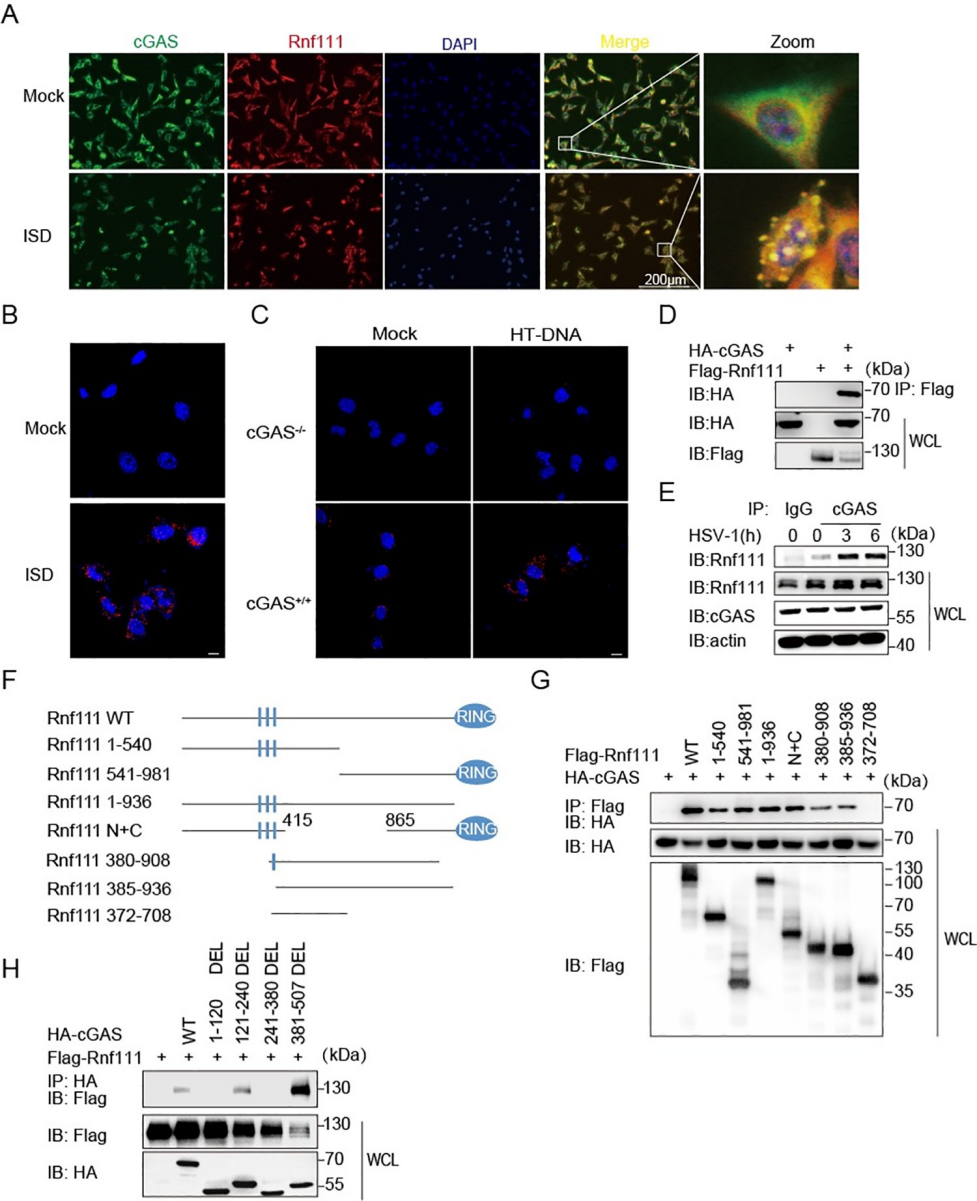
Rnf111 interacts with cGAS. (A) MEFs were stimulated with ISD for 3h, stained with indicated antibodies and imaged by confocal microscopy. Scale bars represent 200 μm. (B) MEFs were stimulated with ISD for 3h, then PLA analysis was applied to detect the interaction between cGAS and Rnf111. Scale bars represent 10 μm. (C) WT or cGAS-/- L929 were stimulated with HT-DNA for 3h, then PLA analysis was applied to detect the interaction between cGAS and Rnf111. Scale bars represent 10 μm. (D) HEK293T cells were transfected with indicated plasmids, 24h after transfection, cell lysis was immunoprecipitated with an anti-Flag antibody and then immunoblotted with indicated antibodies. (E) MEFs were infected with HSV-1 (MOI = 0.5) for the indicated time, cell lysis was immunoprecipitated with an anti-cGAS or IgG antibodies and then immunoblotted with indicated antibodies. (F) Diagrams of Rnf111 truncations used in this paper. (G) HEK293T cells were transfected with indicated plasmids for 24h, then cell lysis was immunoprecipitated with anti-Flag antibody and then immunoblotted with indicated antibodies. (H) HEK293T cells were co-transfected with plasmids of different truncations of cGAS and Rnf111, 24h after transfection, lysates of HEK293T were immunoprecipitated with anti-HA antibody and then immunoblotted with indicated antibodies.
