# Supplementary figures and images for: RNF111-facilitated neddylation potentiates cGAS-mediated antiviral innate immune response
Source: PLoS Pathog. 2021 Mar 15;17(3):e1009401. doi: 10.1371/journal.ppat.1009401 (PMC7959372; doi:10.1371/journal.ppat.1009401)

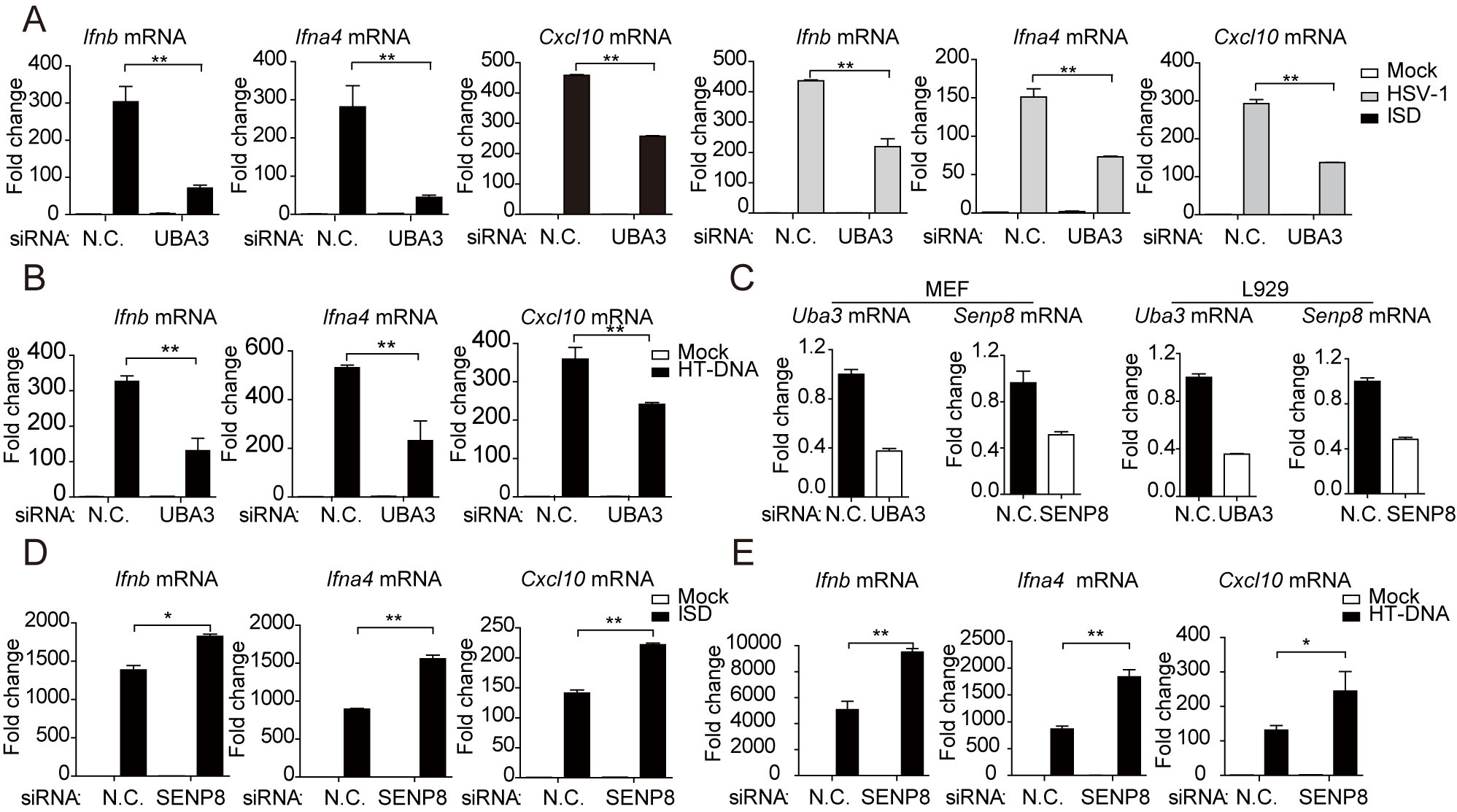

Supplement: S1 Fig — (A) MEFs were transfected with negative control (N.C.) or Uba3 siRNAs for 48h, then stimulated with HSV-1 (MOI = 1) or ISD for 6h, the transcription of Ifnb, Ifna4 and Cxcl10 were measured by qRT-PCR. (B) L929 were transfected with N.C. or Uba3 siRNAs for 48h, then stimulated with HT-DNA for 6h, the transcription of Ifnb, Ifna4 and Cxcl10 were measured by qRT-PCR. (C) MEFs or L929 were transfected with the indicated siRNA, and the Uba3 or Senp8 mRNA was measured by qRT-PCR. (D and E) MEFs and L929 were transfected with N.C. or Senp8 siRNAs for 48h, and then stimulated with ISD or HT-DNA for 6h, the transcription of Ifnb, Ifna4 and Cxcl10 were measured by qRT-PCR, respectively. Graphs are presented as means ± SEM, data are representative of three independent experiments, *P <0.05; **P <0.01 (One-way ANOVAs followed by Tukey’s post hoc test). (TIF) [file ppat.1009401.s001.tif]

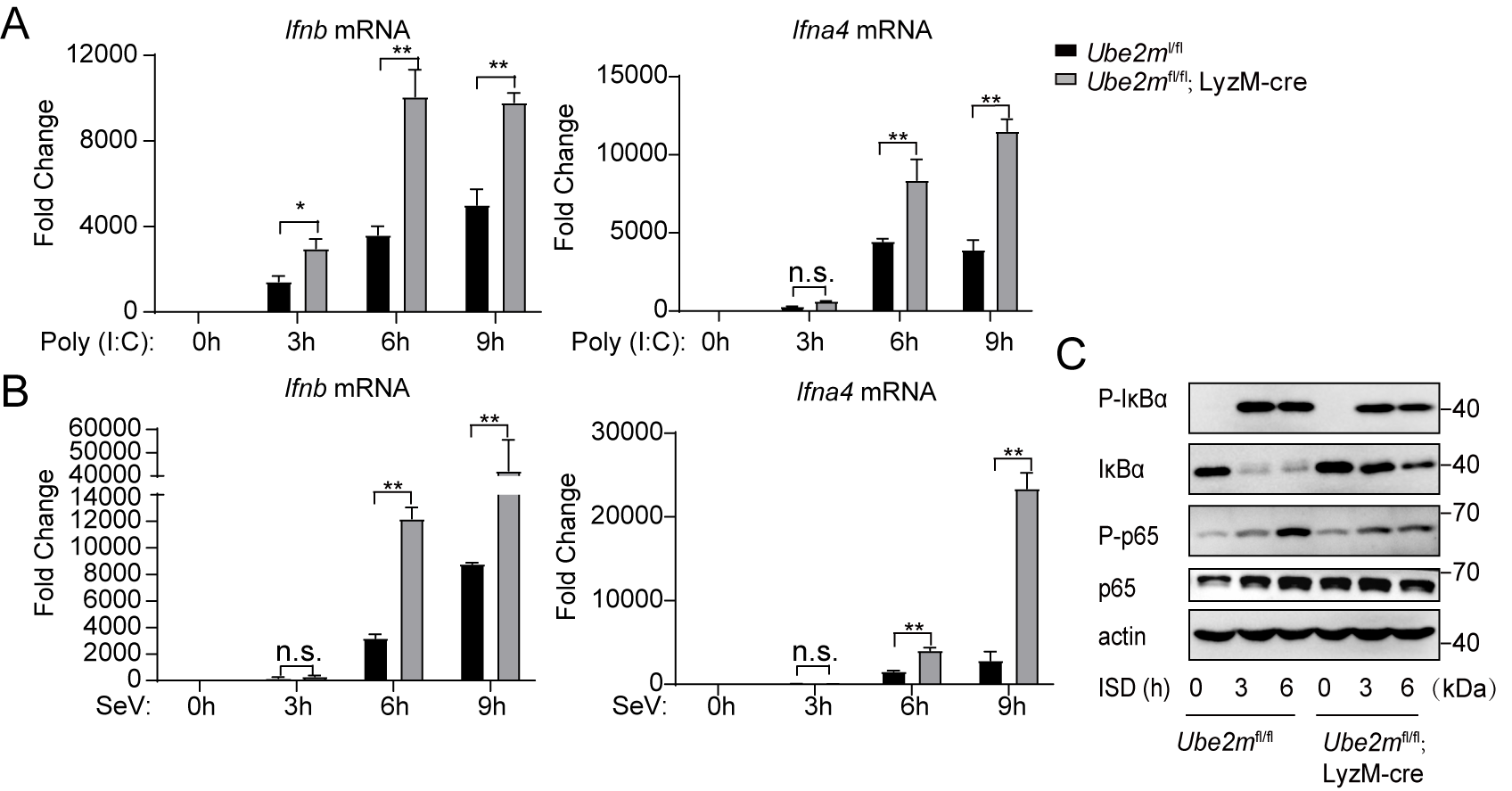

Supplement: S2 Fig — (A) Effects of Ube2m deficiency on the expression of Ifnb and Ifna4 after Poly(I:C) stimulation for indicated hours in BMDM. (B) Effects of Ube2m deficiency on the expression of Ifnb and Ifna4 after SeV (MOI = 1) infection in BMDM. (C) Effects of Ube2m deficiency on the phosphorylation of p65 and IκBα after ISD stimulation for the indicated time. Graphs are presented as means ± SEM, data are representative of three independent experiments, *P <0.05; **P <0.01 (One-way ANOVAs followed by Tukey’s post hoc test). (TIF) [file ppat.1009401.s002.tif]

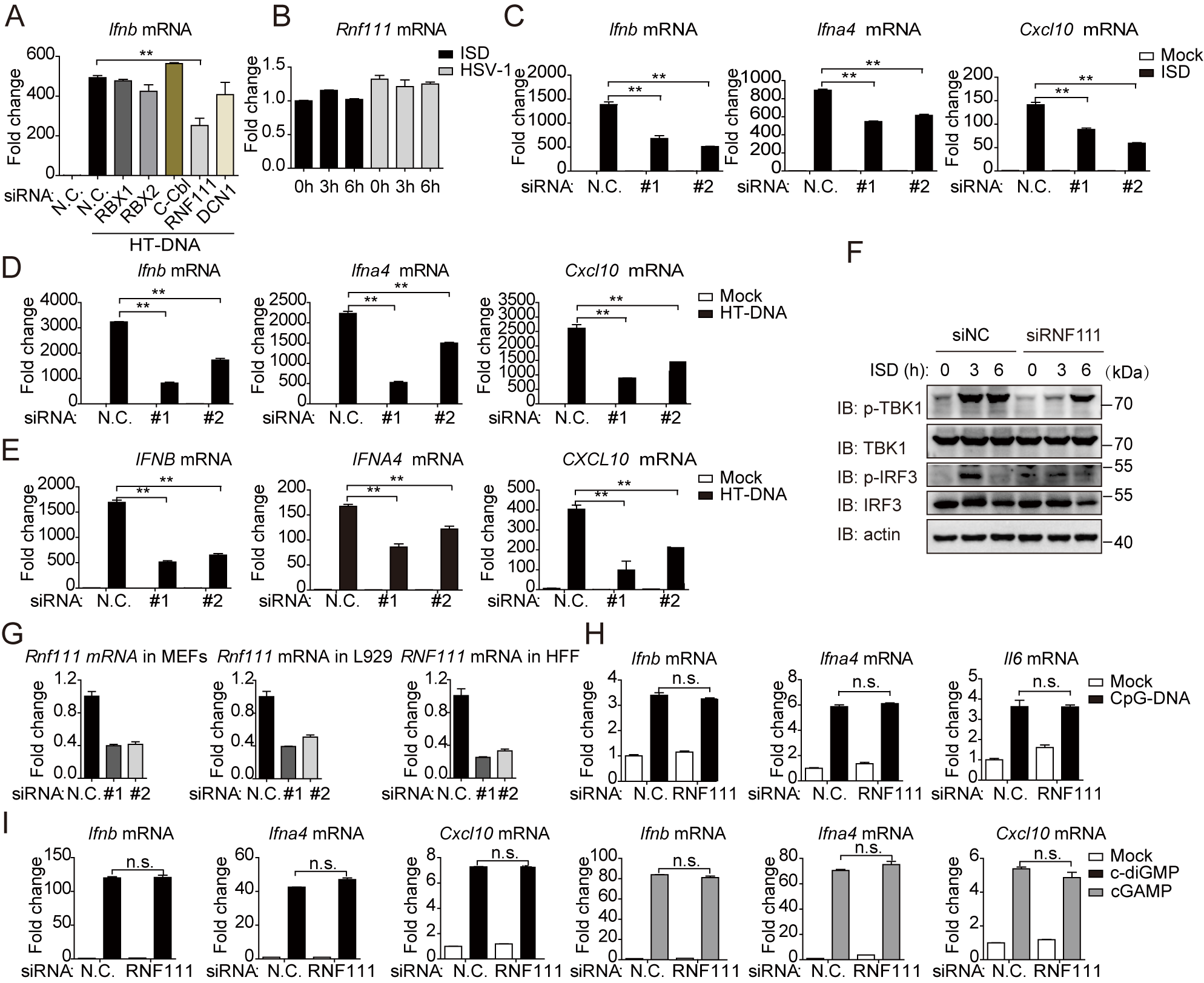

Supplement: S3 Fig — (A) MEFs were transfected with indicated siRNAs for 48h, then stimulated with HT-DNA for 6h, the transcription of Ifnb was measured by qRT-PCR. (B) MEFs were treated with ISD or HSV-1 (MOI = 1) for indicated time, the transcription of Rnf111 was measured by qRT-PCR. MEFs (C), L929 (D) were transfected with N.C. or Rnf111 siRNAs for 48h, and then stimulated with ISD or HT-DNA for 6h, the transcription of Ifnb, Ifna4 and Cxcl10 were measured by qRT-PCR. (E) HFF were transfected with N.C. or RNF111 siRNAs for 48h, and then stimulated with HT-DNA for 6h, the transcription of IFNB, IFNA4 and CXCL10 were measured by qRT-PCR. (F) MEFs were transfected with N.C. or Rnf111 siRNAs for 48h, and then stimulated with ISD for 6h, the phosphorylation of TBK1/IRF3 were analyzed by SDS-PAGE. (G) MEFs (left), L929 (middle) or HFF (right) were transfected with the indicated siRNA, and the RNF111 mRNA was measured by qRT-PCR. (H) MEFs were transfected with N.C. or Rnf111 siRNAs for 48h, and then transfected with CpG-DNA for 6h, the transcription of Ifnb, Ifna4 and Il6 were measured by qRT-PCR. (I) MEFs were transfected with N.C. or Rnf111 siRNAs for 48h, and then stimulated with cGAMP or c-di-GMP for 6h, the transcription of Ifnb, Ifna4 and Cxcl10 were measured by qRT-PCR. Graphs are presented as means ± SEM, data are representative of three independent experiments, *P <0.05; **P <0.01 (One-way ANOVAs followed by Tukey’s post hoc test). (TIF) [file ppat.1009401.s003.tif]

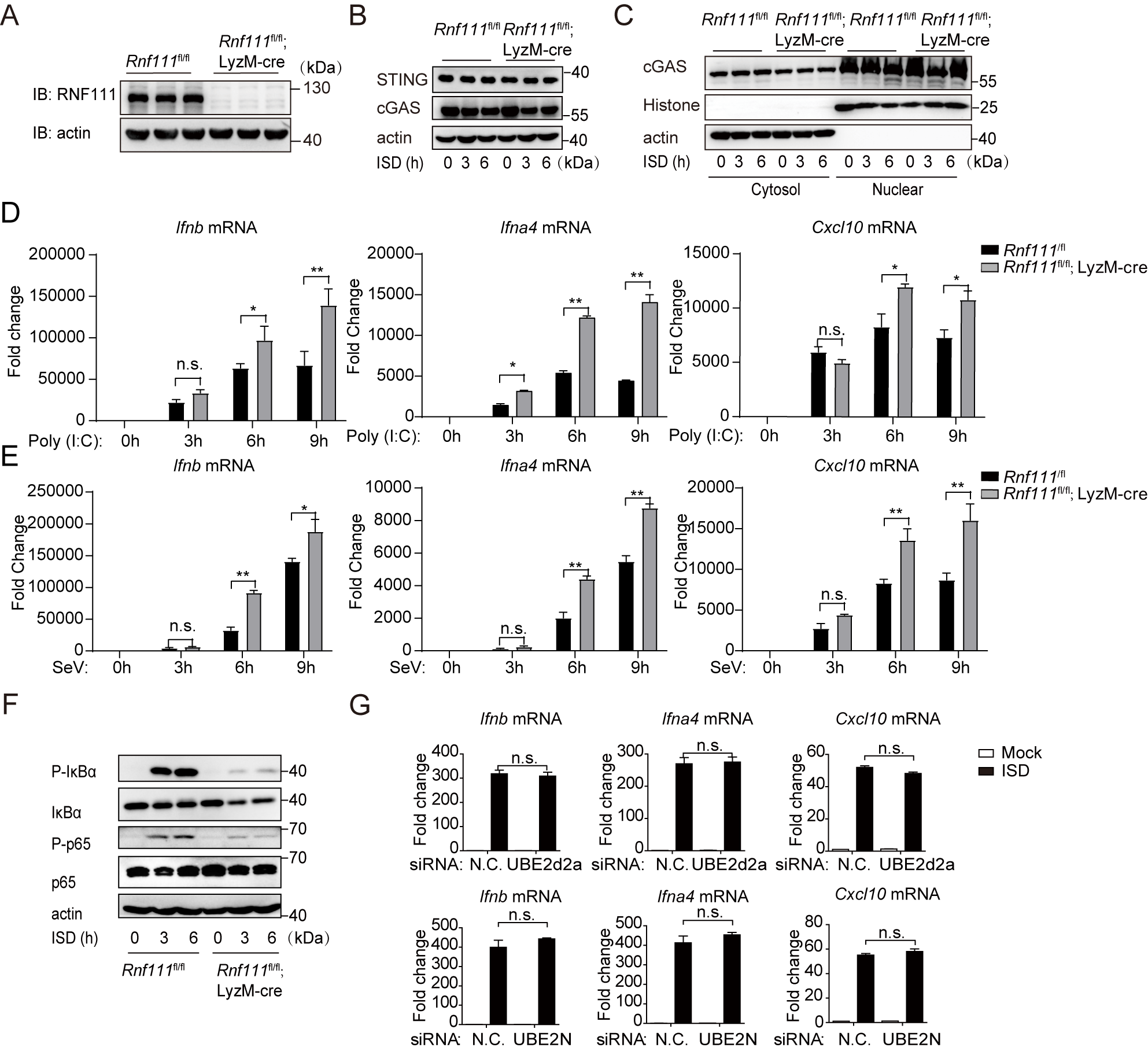

Supplement: S4 Fig — (A) BMDM were harvested from Rnf111fl/fl or Rnf111fl/fl; LyzM-cre mice respectively, the protein level of Rnf111 was confirmed by Western Blot. (B) Effect of Rnf111 deficiency on the protein levels of cGAS and STING after ISD stimulation for indicated time. (C) Effect of Rnf111 deficiency on the protein level of cGAS in the cytoplasmic and nuclear fractions. (D) Effects of Rnf111 deficiency on the expression of Ifnb and Ifna4 after Poly(I:C) stimulation for indicated hours in BMDM. (E) Effects of Rnf111 deficiency on the expression of Ifnb and Ifna4 after SeV (MOI = 1) stimulation in BMDM. (F) Effects of Rnf111 deficiency on the phosphorylation of p65 and IκBα after ISD transfection for the indicated time. (G) MEFs were transfected with N.C., Ube2n or Ube2d2a siRNAs for 48h, and then stimulated with ISD for 6h respectively, the transcription of Ifnb, Ifna4 and Cxcl10 were measured by qRT-PCR. Graphs are presented as means ± SEM, data are representative of three independent experiments, *P <0.05; **P <0.01 (One-way ANOVAs followed by Tukey’s post hoc test). (TIF) [file ppat.1009401.s004.tif]

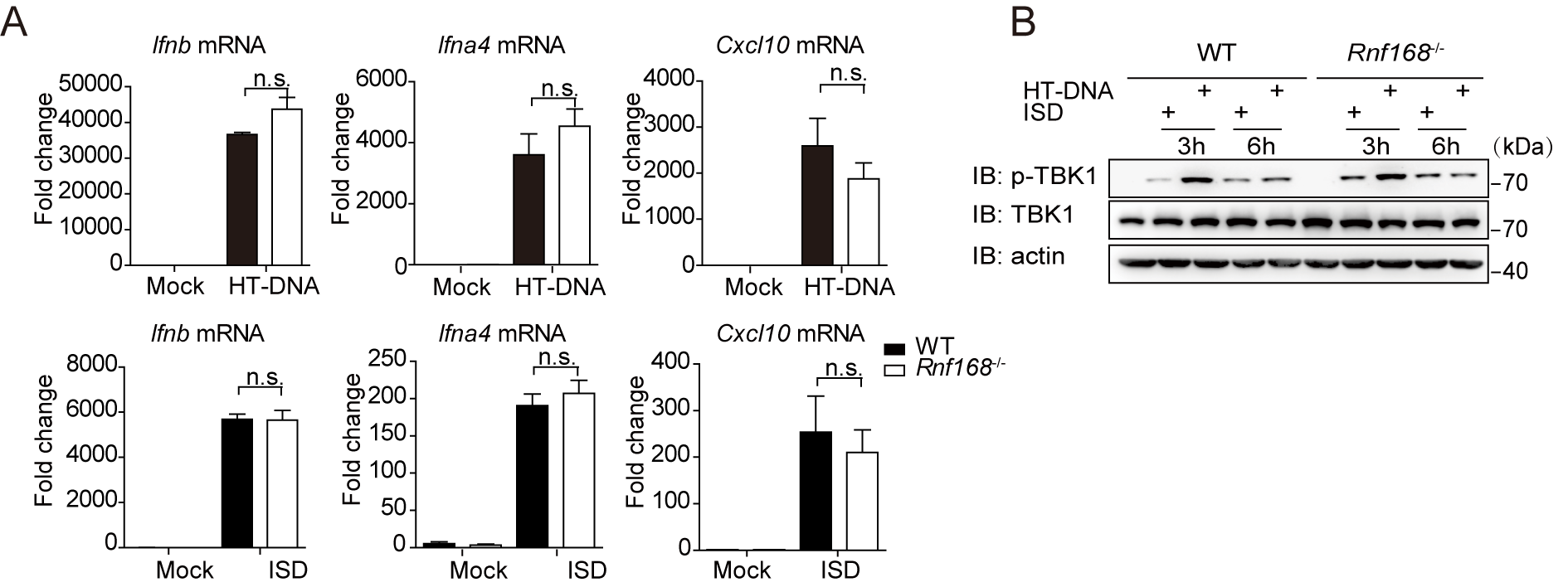

Supplement: S5 Fig — (A) Effect of Rnf168 deficiency on the expression of Ifnb, Ifna4 and Cxcl10 after HT-DNA or ISD stimulation in BMDM. (B) Effects of Rnf168 deficiency on phosphorylation of TBK1 after ISD or HT-DNA stimulation for indicated time. Graphs are presented as means ± SEM, data are representative of three independent experiments, *P <0.05; **P <0.01 (One-way ANOVAs followed by Tukey’s post hoc test). (TIF) [file ppat.1009401.s005.tif]

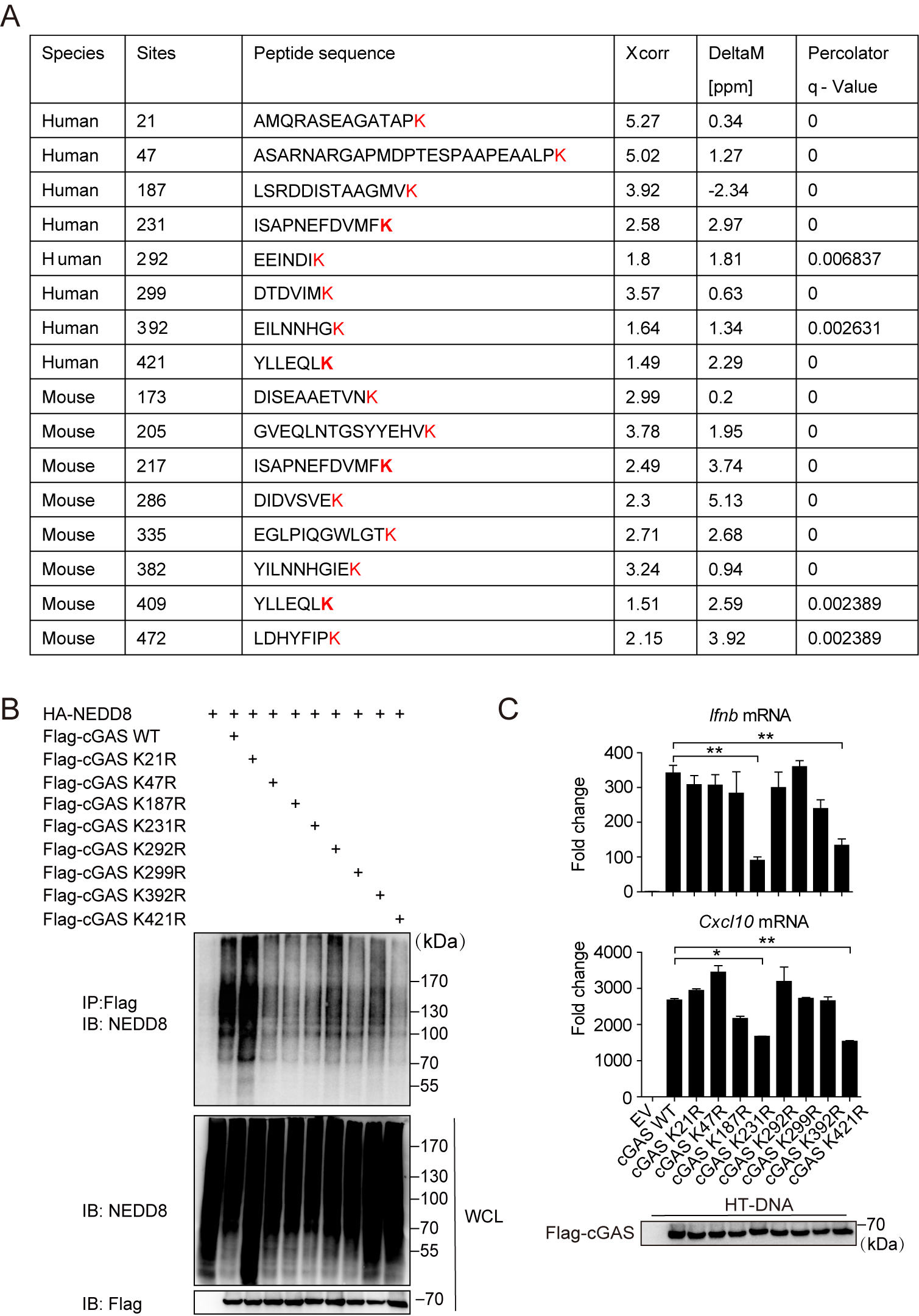

Supplement: S6 Fig — (A) Summary of the identified neddylation sites of cGAS using mass spectrometry data in human and mouse (FDR<0.01). The neddylation modified Lys residues were marked in red, and the conserved Lys residues under neddylation in both human and mouse cGAS were shown in bold and larger font. (B) HEK293T cells were transfected with Flag-cGAS or its K-to-R mutations. At 48h after transfection, cells were subjected to denatured immunoprecipitation with anti-Flag antibody and then analyzed by immunoblotting with indicated antibodies. (C) HEK293 cells were transfected with Flag-cGAS or its K-to-R mutations. At 24h after transfection, cells were stimulated with HT-DNA for 6h, the transcription of Ifnb and Cxcl10 were measured by qRT-PCR. Graphs are presented as means ± SEM, data are representative of at least three experiments, *P <0.05; **P <0.01 (One-way ANOVAs followed by Tukey’s post hoc test). (TIF) [file ppat.1009401.s006.tif]

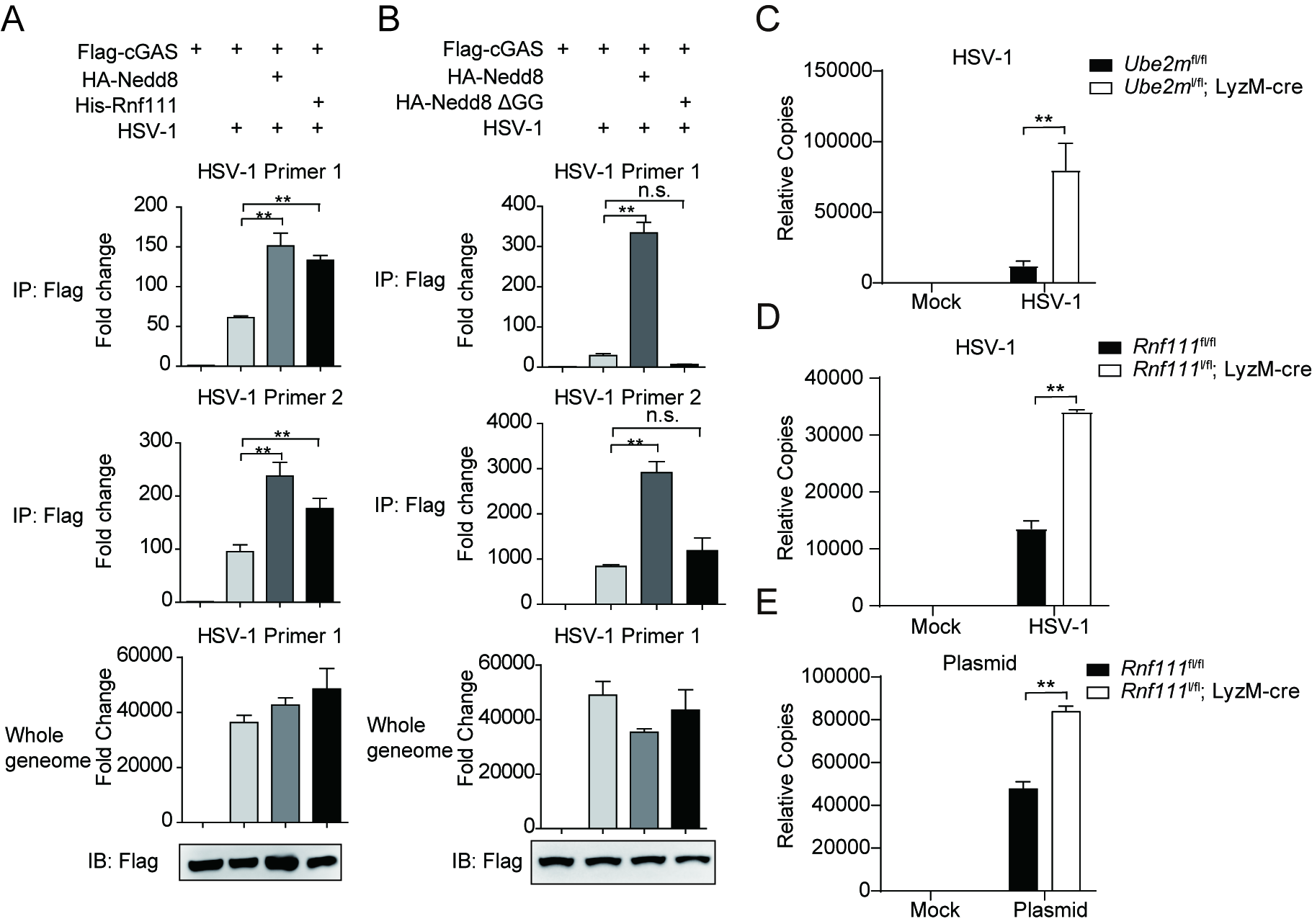

Supplement: S7 Fig — (A) HEK293T cells were transfected with Flag-cGAS, HA-Nedd8 and His-Rnf111, 45h after transfection, cells were stimulated with HSV-1 (MOI = 0.5) for 3h. After adding mouse cDNA as an external reference, cell lysates were immunoprecipitated with anti-Flag antibody, then cGAS-bound DNA was extracted and quantified by qRT-PCR by normalized to mouse GAPDH. (B) HEK293T cells were transfected with Flag-cGAS, HA-Nedd8 and HA-Nedd8ΔGG, 45h after transfection, cells were stimulated with HSV-1 (MOI = 0.5) for 3h. After adding mouse cDNA as an external reference, cell lysates were immunoprecipitated with anti-Flag antibody, then cGAS-bound DNA was extracted and quantified by qRT-PCR by normalized to mouse GAPDH. (C) Ube2m deficiency BMDMs were infected with HSV-1 (MOI = 0.5) for 3h, cells were harvested, then genomic DNA was extracted and the relative copies of HSV-1 were quantified by qRT-PCR by normalized to genomic GAPDH. (D) Rnf111 deficiency BMDMs were infected with HSV-1 (MOI = 0.5) for 3h, cells were harvested, then genomic DNA was extracted and the relative copies of HSV-1 were quantified by qRT-PCR by normalized to genomic GAPDH. (E) Rnf111 deficiency BMDMs were transfected with an empty plasmid for 3h, cells were harvested, then genomic DNA was extracted and the relative copies of plasmid were quantified by qRT-PCR by normalized to genomic GAPDH. Graphs are presented as means ± SEM, data are representative of three independent experiments, *P <0.05; **P <0.01 (One-way ANOVAs followed by Tukey’s post hoc test). (TIF) [file ppat.1009401.s007.tif]

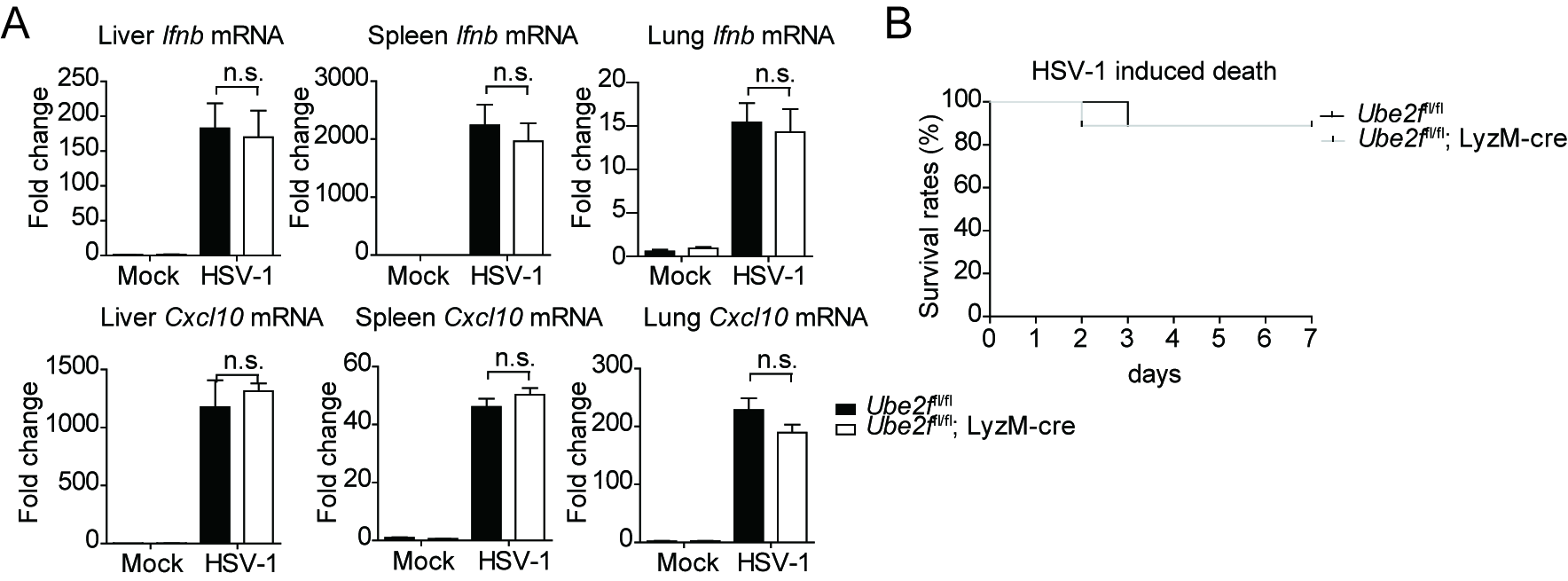

Supplement: S8 Fig — (A) Mice were injected intravenously with HSV-1 (1.5×10^7^ pfu per mouse) for 12h, tissues form Ube2f cKO or the control group were harvest and the relative expression of Ifnb and Cxcl10 in livers, spleens and lungs were measured by qRT-PCR, respectively (n = 6–10). (B) Mice of the Ube2f cKO or the control group were injected intravenously with HSV-1 (6×10^7^ pfu per mouse), and the survival rates were monitored for 7 days (n = 10). Graphs are presented as means ± SEM, data are representative of three independent experiments, *P <0.05; **P <0.01 (One-way ANOVAs followed by Tukey’s post hoc test for A, Mantel-Cox test for B). (TIF) [file ppat.1009401.s008.tif]
