# Supplementary material for: RNF111-facilitated neddylation potentiates cGAS-mediated antiviral innate immune response
Source: PLoS Pathog. 2021 Mar 15;17(3):e1009401. doi: 10.1371/journal.ppat.1009401 (PMC7959372; doi:10.1371/journal.ppat.1009401)
Supplement: S2 Table — (DOCX) [file ppat.1009401.s010.docx]

| Gene | Species | Forward (5’- 3’) | Reverse (5’- 3’) |
| --- | --- | --- | --- |
| *Gapdh* | mouse | GAAGGGCTCATGACCACAGT | GGATGCAGGGATGATGTTCT |
| *Ifnb* | mouse | AGATCAACCTCACCTACAGG | TCAGAAACACTGTCTGCTGG |
| *Ifna4* | mouse | ACCCACAGCCCAGAGAGTGACC | AGGCCCTCTTGTTCCCGAGGT |
| *Cxcl10* | mouse | CGATGACGGGCCAGTGAGAATG | TCAACACGTGGGCAGGATAGGCT |
| *Ifit1* | mouse | CTGAGATGTCACTTCACATGGAA | GTGCATCCCCAATGGGTTCT |
| *Il6* | mouse | TGGGGCTCTTCAAAAGCTCC | AGGAACTATCACCGGATCTTCAA |
| *GADPH* | human | CGGAGTCAACGGATTTGGTC | GACAAGCTTCCCGTTCTCAG |
| *IFNB* | human | ATTGCCTCAAGGACAGGATG | GGCCTTCAGGTAATGCAGAA |
| *IFNA4* | human | TCTTCAGCACAGAGGACTCA | CCTGTATCACACATGCTTCC |
| *CXCL10* | human | GTGGCATTCAAGGAGTACCTC | TGATGGCCTTCGATTCTGGATT |
| *IFIT2* | human | AAGCACCTCAAAGGGCAAAAC | TCGGCCCATGTGATAGTAGAC |
| HSV-1 | HSV-1 | ATACCGACGATCTGCGACCT | TTATTGCCGTCATAGCGCGG |
| HSV-1 | HSV-1 | ACCCACGTACTCCAAGAAGGC | TAAGACCCAAGCATAGAGAGCCA |

Supplementary Table 2. Sequences of primers used in qRT-PCR assay
